# Host-secreted lactate during respiratory viral infection diminishes macrophage antibacterial activity through metabolic reprogramming

**DOI:** 10.1128/iai.00409-26

**Published:** 2026-10-12

**Authors:** Sadia Sultana, Erin Walsh, Jennifer M. Bomberger

**Affiliations:** 1 Department of Microbiology and Immunology, Geisel School of Medicine, Dartmouth College 12285 , Hanover, New Hampshire, USA; University of California Davis, Davis, California, USA

**Keywords:** immunometabolism, lactate, *Pseudomonas aeruginosa*, macrophages, innate immunity, co-infections

## Abstract

In polymicrobial infections, how the host recognizes and responds to pathogens influences which species will persist to cause chronic infections. The human respiratory tract is a common anatomical site for viral-bacterial co-infections, where primary viral infections predispose to secondary bacterial infections, leading to increased morbidity and mortality. Additionally, co-infections are disproportionately prevalent in people with chronic lung diseases, such as chronic obstructive pulmonary disease and cystic fibrosis. We previously reported that primary viral infections and antiviral interferon (IFN) signaling stimulate *Pseudomonas aeruginosa* biofilm formation on airway epithelial cells (AECs). IFN signaling induces aerobic glycolysis in AECs and generates lactate as a cellular byproduct. Given that innate immune systems play an integral role in co-infection dynamics, we investigated the role of host-secreted metabolites (i.e., lactate) on innate immune cell activity during respiratory co-infections. We found that exposure to the apical secretions from IFNβ-treated AECs significantly compromised macrophage antibacterial activity, with the soluble metabolite lactate playing an important role. Macrophages used monocarboxylate transporters (MCTs) and G-protein receptors to transport and/or sense lactate, respectively, and this exposure to lactate diminished their bacterial-killing activity in a time exposure-dependent manner. Lactate exposure particularly reprogrammed macrophage cellular metabolism toward a metabolically uncoupled state by increasing oxidative phosphorylation, fatty acid oxidation, and downregulating the proinflammatory antimicrobial functions. Collectively, these findings provide insights into metabolites as complex regulators of trans-kingdom interactions and epithelial-macrophage crosstalk during respiratory co-infections.

## INTRODUCTION

Complex polymicrobial, cross-kingdom interactions are commonly observed in anatomical sites with diverse microbial communities, such as the gastrointestinal and respiratory tracts (1, 2). Increasing evidence shows that acute viral infections predispose to the acquisition of secondary bacterial infection in those sites (3–5). While the clinical manifestations of viral infections vary from mild to those that require hospitalization, viral-bacterial co-infections often lead to worse outcomes (2, 6, 7). Influenza and respiratory syncytial virus (RSV) are common viral culprits associated with bacterial superinfection in the respiratory tract (8, 9). Particularly, RSV infection is the major cause of infant bronchiolitis and is associated with the development of chronic *Pseudomonas aeruginosa* (PA) infections and pulmonary exacerbation in people with chronic lung diseases, such as cystic fibrosis (CF) (2, 3, 10, 11). PA is a gram-negative opportunistic pathogen, and persistent PA lung infections often lead to severe pulmonary exacerbations, yet the detailed mechanism of how acute viral infections augment the acquisition of chronic PA infections has not been fully elucidated.

The host innate immune system is the first line of defense in both cytokine signaling and pathogen clearance, where macrophages play a pivotal role (12, 13). Macrophages are highly plastic populations; (i) based on their origin, they can be differentiated into tissue-resident or recruited (monocyte-derived) macrophages, and (ii) relying on the environmental cues, they can polarize toward anti-inflammatory or proinflammatory states (14–16). Generally, a proinflammatory state in macrophages is highly phagocytic with antimicrobial and tumoricidal responses, while an anti-inflammatory state promotes macrophages to resolve inflammation, phagocytize apoptotic cells, and promote tissue repair and remodeling. In people with chronic lung diseases like CF or chronic obstructive pulmonary disorder (COPD), recruited macrophages are the predominant macrophage population, and their function to clear infection is impaired (17–19), an effect heightened in a co-infection environment due to impaired macrophage clearance of secondary bacterial infections (18, 20–22).

Using a viral-bacterial co-culture biofilm model in association with airway epithelial cells (AECs), our lab has previously shown that RSV infection and subsequent antiviral response (i) dysregulates iron homeostasis in the airway (2), (ii) releases antiviral extracellular vesicles (EVs) to impair epithelial-macrophage crosstalk (21), and (iii) drives epithelial metabolic reprogramming (23). During RSV infection, interferon signaling is induced and AEC metabolism shifts toward aerobic glycolysis. In a metabolic shift to aerobic glycolysis, cells increase extracellular glucose uptake, which is quickly converted to ATP and produces lactate as a byproduct (23). This metabolic rearrangement is a hallmark of highly proliferative cells (such as cancer cells) and is commonly known as the Warburg effect (24). Lactate is a complex modulator in the tumor microenvironment and has long been considered a waste product. However, recent studies highlight lactate as an energy source, a substrate for post-translational and epigenetic modifications, and a signaling molecule of the innate and adaptive immune cells (25, 26). A key study in tumor research by Colegio et al. demonstrated that tumor cell-generated lactate can signal to or be transported into the local macrophage population, thereby polarizing them toward an immunosuppressive (anti-inflammatory) state (27).

Our observation of increased aerobic glycolysis and lactate secretion in virus-infected AECs highlights surprising similarities between metabolic remodeling during viral infection and the metabolic setting of the tumor microenvironment. It also suggests that host-derived lactate in the respiratory milieu can dysregulate immune (i.e., macrophage) responses during co-infection by preemptively skewing them toward a suppressive state, reducing their ability to clear secondary bacterial infections, the mechanistic details of which have not been investigated. Leveraging our polarized respiratory epithelial model (2, 21) in this work, we sought to determine whether host-secreted soluble metabolites (i.e., lactate) can alter naïve macrophage physiology in the local co-infected environment. Our study reveals that lactate-driven immunometabolic regulation of macrophages during respiratory viral infection favors the acquisition of secondary bacterial infection.

## RESULTS

### Host-secreted metabolites drive macrophage dysfunction during respiratory viral-bacterial co-infections

A hallmark of viral-bacterial co-infections is antiviral signaling leading to impaired antibacterial defense, where macrophage dysfunction plays a vital role (1, 11, 23). We investigated if antiviral interferon signaling in the respiratory epithelium triggers the secretion of factors that impair macrophage antibacterial activity. Previously, our lab showed that primary viral infections and the subsequent antiviral interferon response reprogram airway epithelial metabolism, leading to the secretion of host metabolites in the respiratory milieu (23). To investigate the role of host-secreted metabolites during primary viral infection in regulating antibacterial activity of innate immune cells, we treated macrophages with respiratory epithelial secretions and evaluated macrophage bacterial killing efficiency using an antibiotic protection-based killing assay (21, 28). Polarized respiratory epithelial cells CFBE41o- (human bronchial epithelial cell line derived from an F508del/F508del homozygous cystic fibrosis patient tissue; hereafter AECs) were treated with interferon-β (IFNβ) for 18 h to mimic the host antiviral response, and apical secretions were collected (Fig. 1A). Monocytes isolated from the peripheral blood mononuclear cells of healthy human donors were cultured with M-CSF for 8 days to generate monocyte-derived macrophages (MDMs). MDMs were incubated for 60 min with AEC apical secretions and then challenged with the respiratory pathogen *Pseudomonas aeruginosa* (MPAO1) at a multiplicity of infection of 10. Using bacterial uptake and survival CFUs (see Materials and Methods), we next evaluated MDMs’ bacterial killing efficiency. We observed that MDMs incubated with the apical secretions from IFNβ-treated AECs are significantly impaired in MPAO1 killing as compared to untreated AEC secretions (Fig. 1B). Previously, our lab has reported that antiviral interferon signaling triggers AEC metabolic reprogramming and produces metabolic byproducts, such as lactate, which are secreted into the apical milieu (23). In the current study, we asked if these secreted metabolites can alter macrophage antibacterial activity. We isolated the soluble metabolites from interferon-treated AECs and incubated them with macrophages before performing the killing assay. Interestingly, we

**Fig 1:**
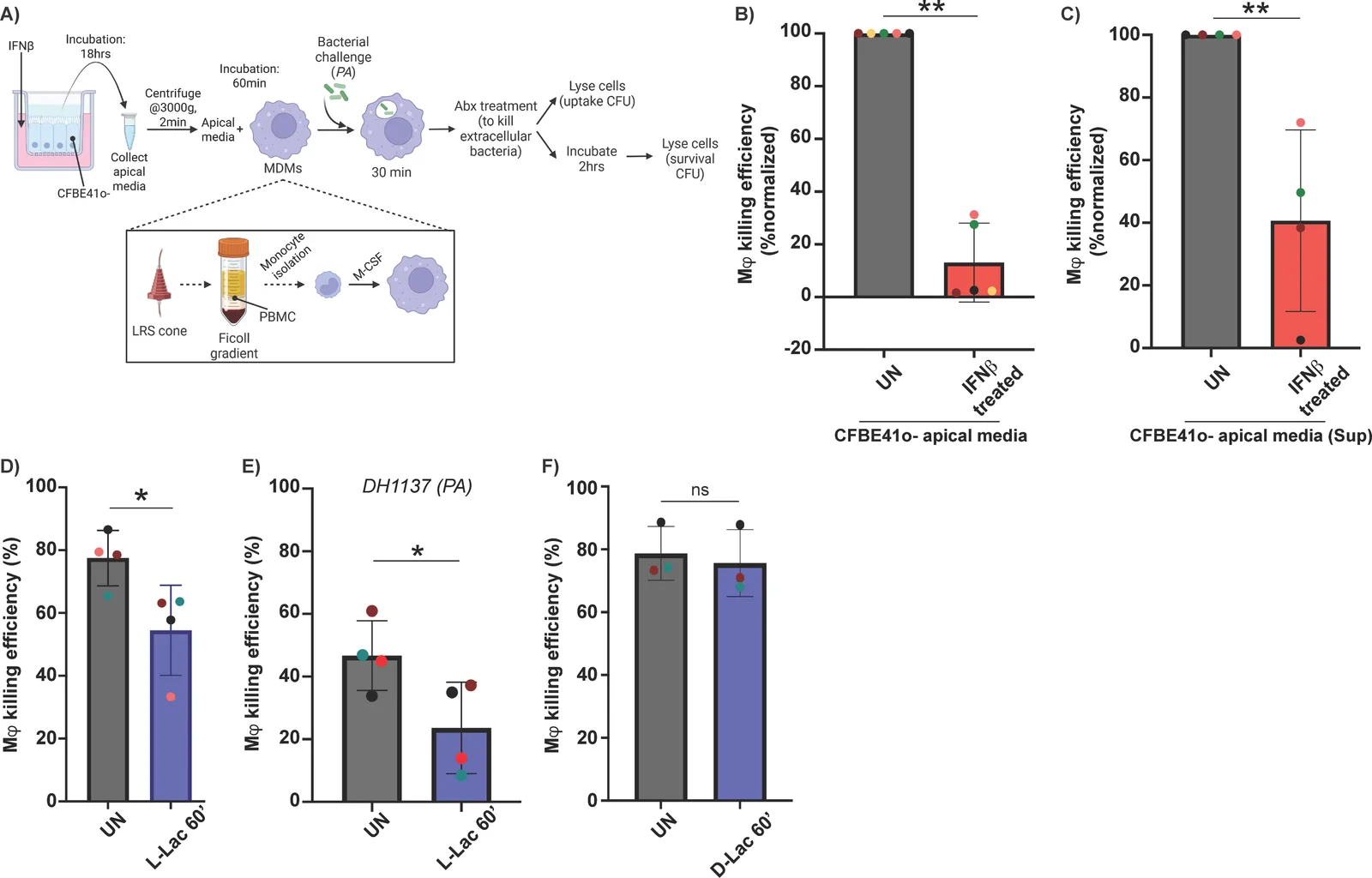
Host-secreted metabolites drive macrophage dysfunction during respiratory co-infections. (**A**) Experimental workflow to generate MDMs, to collect CFBE41o- apical medium after IFNβ treatment, and killing assay using respiratory pathogen MPAO1. Assay result displayed as macrophage (Mφ) killing efficiency (**B**); data are normalized to the untreated (UN) condition (apical medium without IFNβ treatment). (**C**) Apical medium from panel A was ultracentrifuged to separate the soluble supernatant (Sup) fraction and used to analyze Mφ killing efficiency; data are normalized to the UN condition (apical medium supernatant). Killing assays were performed after Mφ were incubated with (**D**) 10 mM L-lactate, (**E**) *Pseudomonas aeruginosa* clinical isolate DH1137 after exposure to 10 mM L-lactate, and (**F**) 10 mM D-lactate for 60 min. Macrophage killing efficiency (%) was calculated as described in Materials and Methods, where each color denotes a donor. Statistics: linear mixed-effects model, with donor as a random effect and treatment as a fixed effect. *P < 0.05; **P < 0.01; ns, not significant.

observed a similar reduction in MDM killing efficiency when incubated with the secreted metabolites (soluble supernatant fraction) from IFNβ-treated AECs (Fig. 1C). Together, these results are consistent with the conclusion that secretory soluble metabolites in the apical milieu during primary respiratory viral infection can regulate macrophage anti-bacterial activity.

### Host-secreted lactate during viral infection reduces macrophage antibacterial activity

Viral infections are known to trigger metabolic remodeling in AECs, including increased levels of aerobic glycolysis (23, 29, 30). L-lactate is a by-product of aerobic glycolysis and is present in the infection microenvironment (23, 24, 26, 31–33). It is well established in the cancer biology literature that L-lactate present in the tumor microenvironment can act as a signaling molecule and/or energy source for neighboring immune cells and skew macrophage polarization toward an anti-inflammatory state (24, 25, 34, 35). Therefore, we sought to explore whether lactate present in apical secretions of virus-infected AECs contributes to MDMs’ reduced bacterial-killing efficiency. We incubated MDMs with 10 mM L-lactate, which approximates the lactate concentration during respiratory viral infection (23) and performed the killing assay. MDMs’ bacterial-killing efficiency was significantly reduced after 60-min incubation with lactate (Fig. 1D), but not after 24-h incubation (Fig. S1A), suggesting that MDMs exhibit a time-dependent response to lactate. To determine whether the observed phenotype is a bacterial strain-specific phenotype, we performed the assay using cystic fibrosis lung PA isolate DH1137 (36). Consistent with the findings with the PAO1 strain of PA, MDMs showed similar defects in DH1137 killing efficiency (Fig. 1E). Lactate can exist in two isomeric forms, L-lactate and D-lactate; L-lactate is mostly produced by host cells and D-lactate by bacterial species, while some *Lactobacillus* species and *Staphylococcus aureus* can generate L-lactate (37, 38). To investigate if MDMs respond similarly to D-lactate, we carried out the killing assays after D-lactate exposure, and the data showed no reduction in MDMs’ killing efficiency (Fig. 1F). Finally, we exposed MDMs to a high concentration of L-lactate that resembles the lactate level in the tumor microenvironment (39) and performed the killing assay to examine if accumulation of lactate can exhibit a similar response. Interestingly, we found that exposure to 30 mM L-lactate for 60 min did not skew macrophage antibacterial activity (Fig. S1B). Together, our experiments support the conclusion that L-lactate (hereafter referred to as lactate) negatively impacts macrophage antibacterial activity in an exposure time- and dose-dependent manner.

### Macrophages exposed to lactate show a defect in bacterial killing

Macrophage killing efficiency {[(avg. CFU uptake − avg. CFU survival) / avg. CFU uptake] × 100%} quantifies how efficiently a macrophage kills the bacteria it has phagocytosed. Based on the findings that lactate treatment reduced macrophage antibacterial activity, we asked whether this phenotype was due to a decrease in bacterial uptake, killing, or both (40, 41). Comparison between bacterial uptake and survival CFU counts, mainly how many bacteria are able to survive from what has been taken up by the macrophages, suggests a deficiency in MDMs’ bacterial-killing activity rather than in bacterial uptake (Fig. 2A and B). Additionally, we performed macrophage uptake assays using fluorescently tagged (i) *Pseudomonas aeruginosa* strain PAO1-tdTomato and (ii) latex beads. Macrophages were treated with lactate, challenged with latex beads or PAO1-tdTomato for 30 min, and analyzed by flow cytometry to quantify the bead- or bacteria-positive macrophage population. Our data showed similar levels of beads or bacterial uptake in both the lactate-treated and untreated conditions (Fig. 2C and Fig. S2A and B). In summary, these findings demonstrate that lactate impairs macrophage bacterial clearance efficacy rather than pathogen uptake.

**Fig 2:**
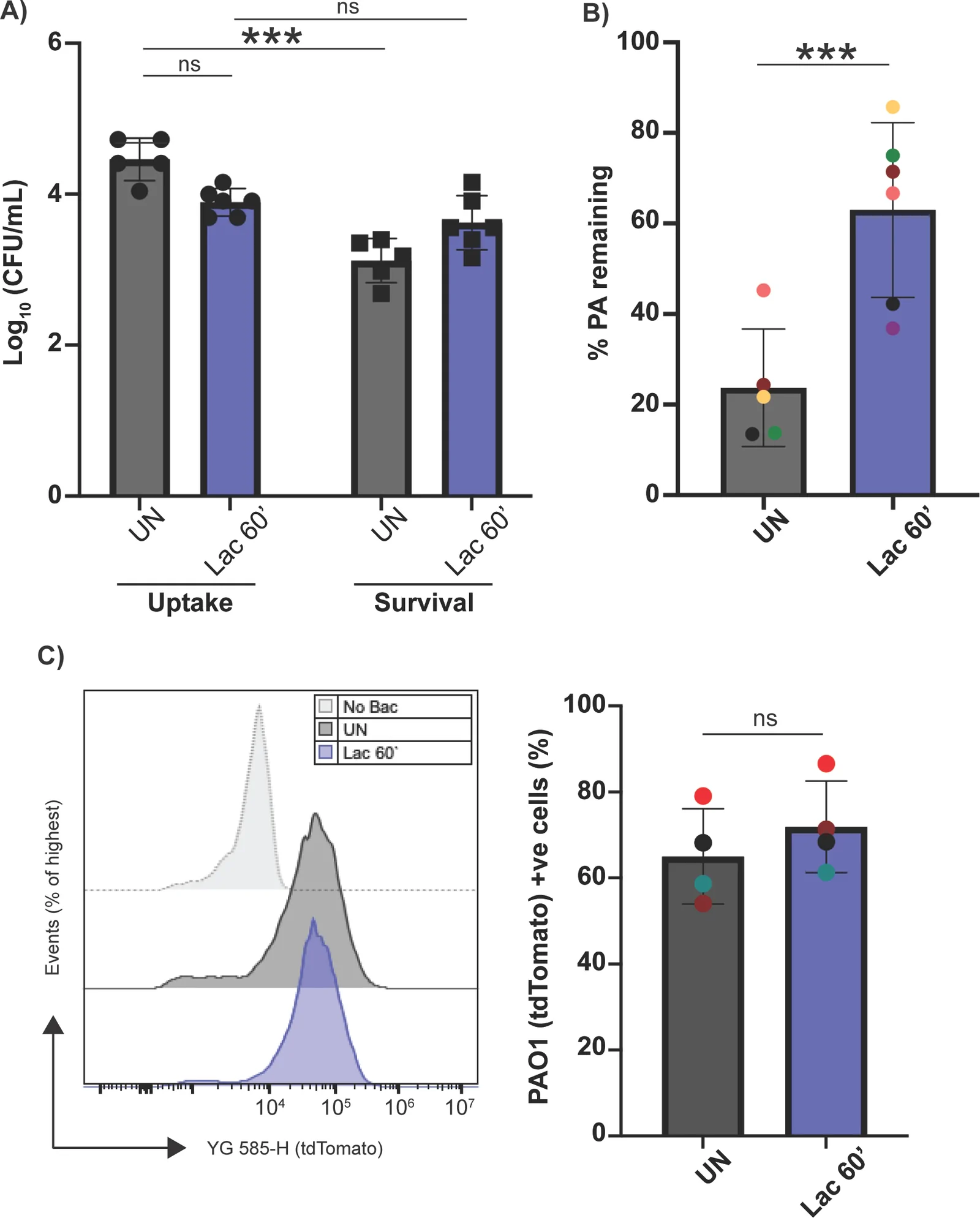
Lactate diminishes macrophage antibacterial activity. (**A**) Mφ bacterial (PA) uptake and survival count from the killing assay in the lactate-treated condition or untreated control. (**B**) The capability of Mφ to clear PA was evaluated by quantifying %PA remaining using the uptake and survival CFUs from panel A. %PA remaining represents the number of bacteria that survived 2 h post-infection compared to time 0 (see Materials and Methods). Each color denotes a donor. (**C**) Histogram of fluorescent PAO1-tdTomato uptake by Mφ in lactate-treated and untreated conditions. Mφ without bacterial exposure was used as a control. The bar graph shows the percentage of Mφ cells positive for PAO1-tdTomato. At least 10,000 events were analyzed per condition using the CytoFlex flow cytometer. Each color denotes a donor. Statistics: linear mixed-effects model, with donor as a random effect and treatment as a fixed effect. ***P < 0.001; ns, not significant.

### Lactate present in the co-infection airway lumen signals through pathways similar to those in the tumor microenvironment

The conventional view of metabolites as merely a “fuel or energy” source to support metabolic pathways has been challenged over the last decade. It is now established that many metabolites can function as intracellular or extracellular signaling molecules to regulate the activity of different enzymes and cellular pathways (42–44). In hypoxic or acidic environments, such as in tumor cells, lactate is transported into the cell via the monocarboxylate transporters MCT1 and MCT4 (35, 45) and also sensed by membrane receptors (G protein-coupled receptors [GPCRs]; GPR81/GPR132) (46, 47). To test whether lactate present in co-infection settings signals through similar GPCRs and MCTs, we performed the killing assay using specific GPCR and MCT inhibitors (Fig. 3A), as reported in literature (33, 48). Pertussis toxin (PTX), a bacterial AB5 toxin, inhibits Gαi/o G protein signaling cascades (49–51), a pathway used by the GPR81 and GPR132 for intracellular signaling (46, 47). We incubated macrophages with PTX for 18 h, followed by 60-min lactate treatment and MPAO1 challenge (Fig. 3A). The presence or absence of PTX did not affect macrophage killing efficiency in the lactate-free condition; however, in the lactate condition, PTX restored macrophage killing efficiency to levels similar to those in the untreated condition (Fig. 3B). Consistent with this idea, we also treated macrophages with 2-cyano-3-(4-hydroxyphenyl)−2-propenoic acid (CHC), an inhibitor of MCT1 and MCT4. Combined treatment with CHC and lactate also restores macrophage killing efficiency to levels comparable to those of untreated macrophages (Fig. 3C). Together, these data suggest that both GPCR-mediated lactate signaling and MCT-mediated lactate transport suppress macrophage antibacterial activity during respiratory co-infection, consistent with recent literature observation (33).

**Fig 3:**
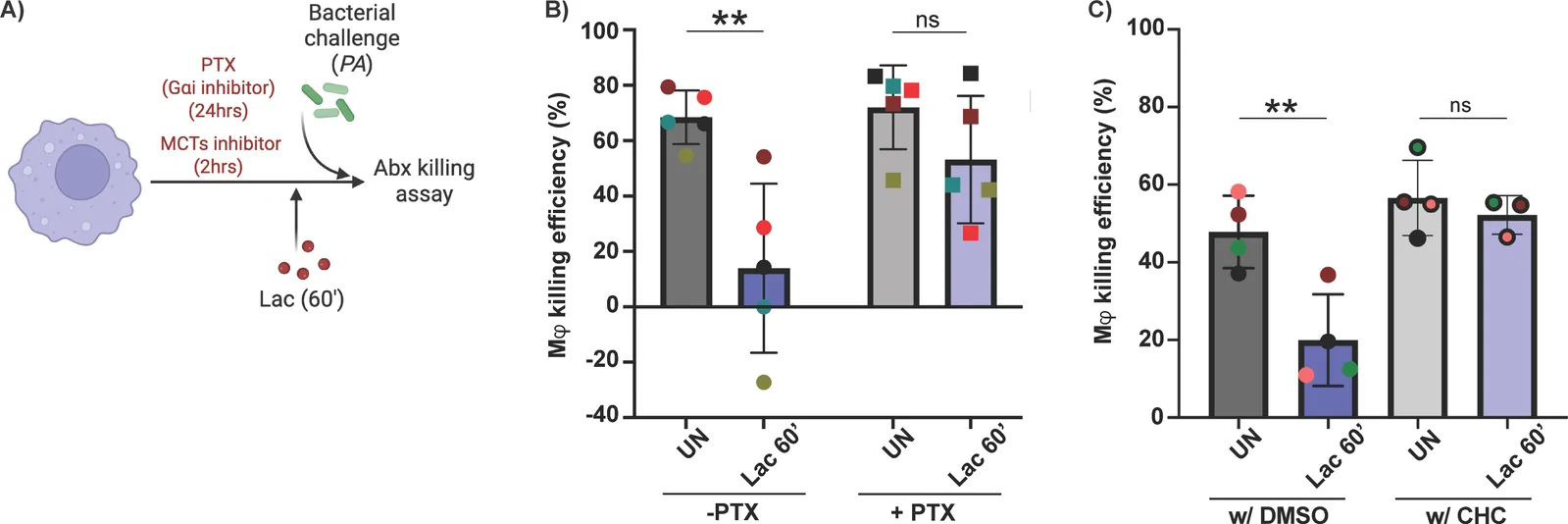
Lactate present in the co-infection setting signals through similar MCTs and G-protein pathways as the tumor microenvironment. Killing assay was performed following treatment with Gαi pathway inhibitor pertussis toxin (PTX, 100 nM) for 24 h or MCT-1 and MCT-4 inhibitor α-cyano-4-hydroxycinnamic acid (CHC, 50 nM) for 2 h (**A**). Data are visualized as Mφ killing efficiency; both (**B**) PTX and (**C**) CHC incubation restore Mφ killing efficiency to the untreated level. Each color denotes a donor. Statistics: linear mixed-effects model, with donor as a random effect and treatment as a fixed effect. *P < 0.05; **P < 0.01; ns, not significant.

### Brief exposure to lactate does not influence macrophage canonical proinflammatory pathways

Next, we sought to characterize the interaction dynamics between lactate and macrophages and assessed the physiological features of macrophages after lactate exposure. At first, to trace the possible cytotoxic effect of lactate on macrophages, we performed the glucose-6-phosphate dehydrogenase release assay (52) and found that 10 mM lactate exposure is not cytotoxic to macrophages at either short or long time points (Fig. S3A). Macrophages defective in antibacterial activity are generally referred to as anti-inflammatory macrophages and often exhibit elongated morphology (47). Microscopic analysis of lactate-treated macrophages showed morphological features similar to those of untreated macrophages (Fig. S2C), suggesting that the negative effect of lactate on macrophage bacterial clearance activity is not dependent on morphological changes.

To investigate the classical anti-inflammatory features of macrophages after lactate exposure, we analyzed the changes in cytokine secretion, cellular marker expression, and antimicrobial oxidant production. However, it is important to acknowledge that, while macrophages are generally classified as proinflammatory (highly microbicidal) and anti-inflammatory (resolving) states, *in vivo* they are highly plastic populations that exhibit functional/physiological diversity in response to local environmental cues (14, 53–55). MDMs were treated with lactate, supernatants were collected at different time points during MPAO1 incubation, and the cytokine levels were analyzed using DuoSet ELISA. In both untreated and lactate-treated conditions, proinflammatory cytokine TNFα and anti-inflammatory cytokine IL-10 levels remained similar following 30 min and 2 h of MPAO1 infection (Fig. 4A). Next, we evaluated the expression of anti-inflammatory intracellular (Arg1) and cell surface (CD163 and CD206) markers after 60 min of lactate treatment. Macrophages were stained with marker-specific antibodies and subjected to flow cytometry to quantify the positive cell population. Consistent with the cytokine data, no changes in cell marker staining were found between lactate-treated and untreated macrophages, either when assessed by positive cell staining or mean fluorescence intensity on the positive cells (Fig. 4B and Fig. S4). Reactive oxygen species (ROS) production and phagolysosomal acidification are important features of macrophage bacterial clearance efficacy (56, 57); therefore, we quantified the ROS level and lysosomal pH of lactate-exposed MDMs. We stained MPAO1-infected macrophages with CM-H2DCFDA (to detect ROS) and LysoTracker (to detect lysosomes) and analyzed for positive cell population using flow cytometry. The mean fluorescence intensity of the reporter dyes was similar for both the lactate-treated and untreated conditions (Fig. 4C and D). Taken together, these data suggest that lactate impairs macrophages’ antibacterial activity, though it does not induce changes in conventional polarization state-specific features—a further confirmation that dynamic plasticity exists in macrophages even within the anti-inflammatory state.

**Fig 4:**
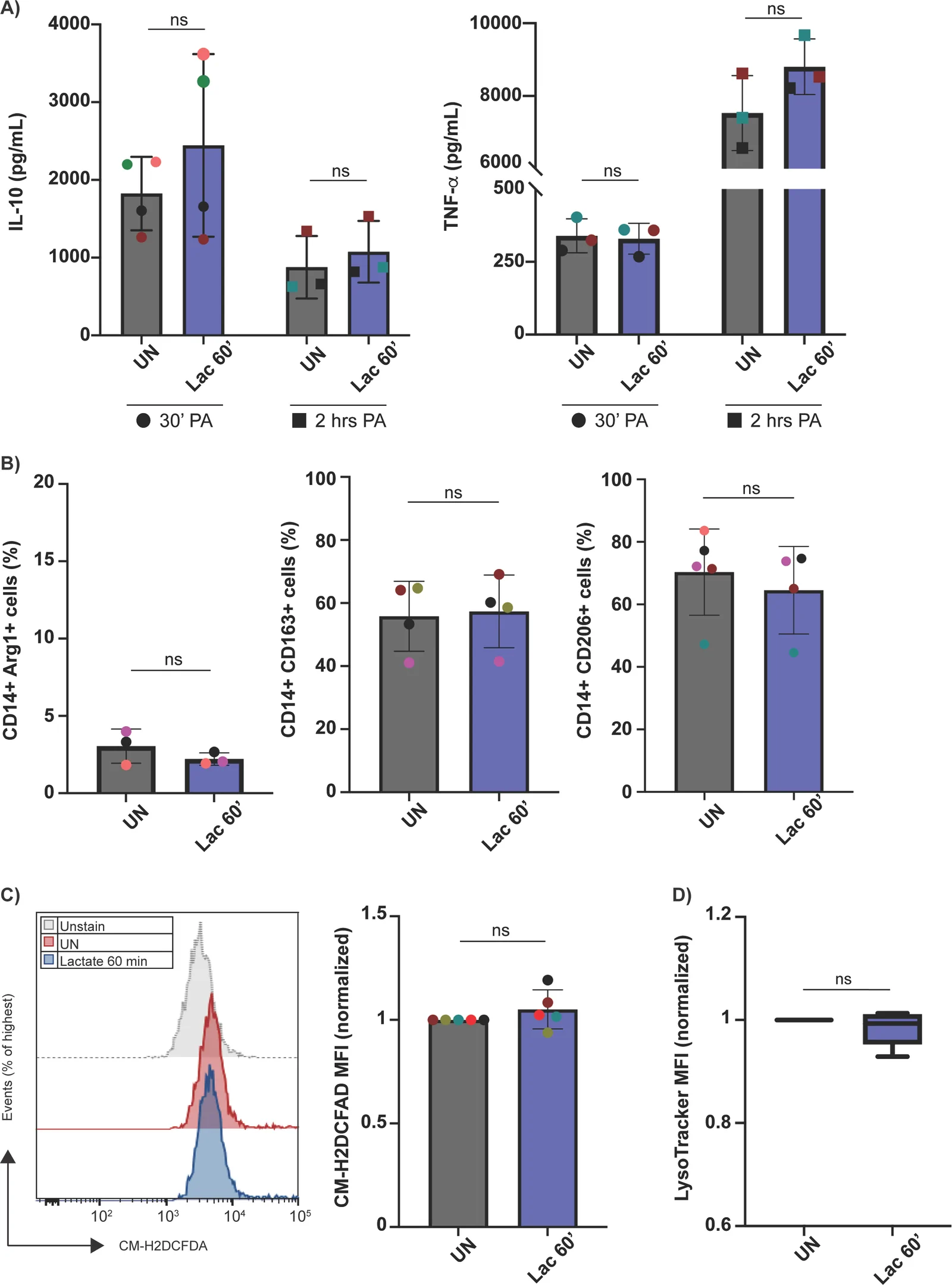
Lactate does not impact macrophage canonical proinflammatory pathways. (**A**) Mφ with or without 10 mM lactate treatment were challenged with MPAO1 for 30 min or 2 h, and the supernatant was collected for cytokine analysis using the DuoSet ELISA kit. IL-10 and TNFα levels were quantified using the manufacturer-supplied standards. (**B**) Mφ cell surface (CD163 and CD206) and intracellular (Arg1) marker expression in control or 10 mM lactate treatment condition were analyzed after incubation with a specific fluorescent antibody for 30 min. Extracellular CD14 was used as an Mφ-specific marker. At least 10,000 events were analyzed per condition using the CytoFlex flow cytometer, and Mφ positive for CD14 and respective markers were analyzed using FlowJo software. Mφ were incubated with 10 mM lactate, challenged with MPAO1, stained with (**C**) CM-H2DCFDA for ROS or (**D**) lysotracker-DeepRed for lysosomal pH, and analyzed using a CytoFlex flow cytometer. At least 10,000 events were analyzed per condition. (**C**) The bar graph shows the mean fluorescent intensity (MFI) of lactate-treated Mφ, normalized to the untreated control. The histogram shows a representative data set. (**D**) MFI was calculated after normalization to the untreated control. Each color denotes a donor. Statistics: linear mixed-effects model, with donor as a random effect and treatment as a fixed effect. ns, not significant.

### Lactate modulates macrophage cellular metabolism

Emerging studies are now highlighting the interconnection between macrophage metabolism, polarization states, and subsequent functional activity in the infection environment (55, 58–60). Proinflammatory macrophages shift their metabolism toward aerobic glycolysis upon activation, and their TCA cycle is also attenuated to facilitate increased ROS production. In contrast, anti-inflammatory macrophages mostly rely on oxidative phosphorylation (OXPHOS) or fatty acid oxidation (FAO) for their energy source (61). Therefore, we next investigated macrophage metabolic state in the presence of lactate. To test for metabolic changes, we performed mitochondrial oxygen consumption and fatty acid oxidation assays.

To evaluate the real-time metabolic state of macrophages after lactate exposure, we performed mitochondrial stress test using the fluorescence-based MitoXpress Xtra oxygen consumption rate (OCR) and Seahorse assays. For the MitoXpress Xtra OCR assay, MDMs were treated with lactate for 30 min, 60 min, or 24 h before obtaining the time-resolved fluorescence reading for 90 min. MDM OCR was significantly increased after shorter (30/60 min) lactate exposure, but not after longer (24 h) incubation (Fig. 5A and Fig. S5). This finding is consistent with our previous killing assay results, in which we observed a similar time-course of macrophage antibacterial activity impairment (Fig. 1D and Fig. S1A). To further investigate the macrophage metabolic profile, we conducted a Seahorse cell Mito Stress assay using the XFe96 Bioanalyzer after sequential addition of specific modulators. This assay measures macrophage baseline respiration (basal OCR), respiration after inhibiting ATP synthesis (proton leak), and forcing mitochondria to work at the maximum capacity (maximum respiration). Assay data revealed that the difference in OCR after lactate exposure is mostly driven by an increase in basal oxygen consumption and proton leak (Fig. 5B), while the maximum respiration showed an increasing trend but statistically nonsignificant.

**Fig 5:**
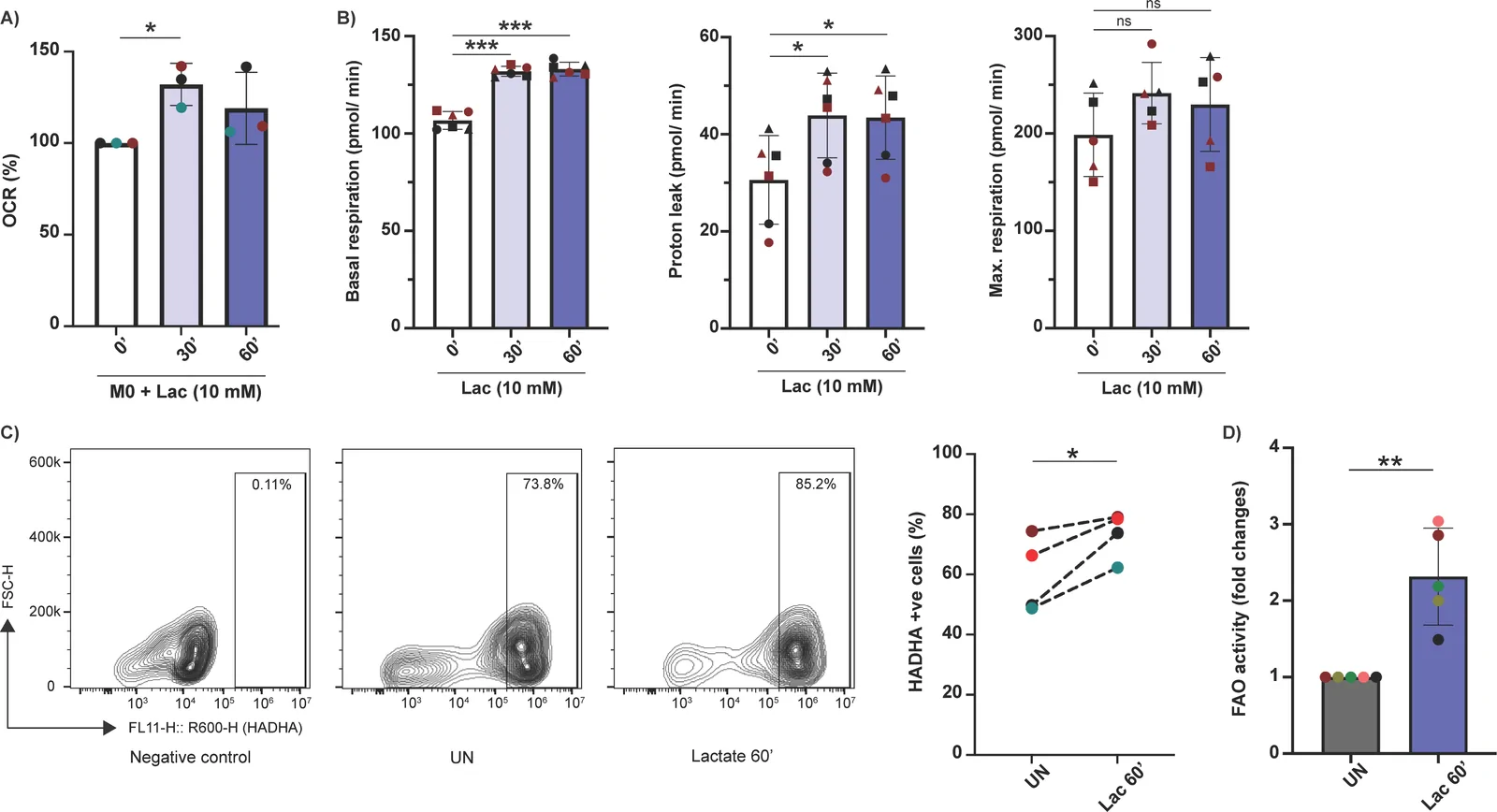
Lactate modulates macrophage cellular metabolism toward an anti-inflammatory-like state. (**A**) Mφ OCR was spectrophotometrically quantified using MitoXpress Xtra probe after exposure to 10 mM of lactate for 30 and 60 min. (**B**) Mφ was treated with 10 mM lactate, and basal respiration, proton leak, and maximum respiration were quantified using Seahorse XF Cell Mito Stress test. Each color denotes a donor, and shape represents technical replicates for each donor. (**C**) Mφ was treated with lactate, and the mitochondrial fatty acid β-oxidation pathway enzyme HADHA level was quantified using flow cytometry. Contour plot of representative data sets, and the graph shows Mφ population positive for HADHA. At least 10,000 events were analyzed per condition. (**D**) FAO activity of Mφ cell lysates, with or without lactate treatment, was quantitatively measured using octanoyl-CoA substrate (see Materials and Methods for details). Each color denotes a donor. Statistics: linear mixed-effects model, with donor as a random effect and treatment as a fixed effect. *P < 0.05; **P < 0.01; ***P < 0.001; ns, not significant.

Anti-inflammatory macrophages also exhibit changes in lipid metabolism with an increase in mitochondrial fatty acid β-oxidation (FAO) (62, 63). Long-chain fatty acids are imported into the mitochondrial membrane, where they are oxidized by mitochondrial dehydrogenases, such as ACADVL (catalyzes the first step of the β-oxidation pathway) and HADHA/B (enzymes for the final three steps of β-oxidation), generating acetyl-CoA, NADH, or FADH2. These fuel the TCA cycle and the electron transport chain to generate ATP (55, 64). Therefore, as a secondary confirmation of macrophage metabolic remodeling after lactate exposure, we analyzed (i) the level of expression of a mitochondrial fatty acid β-oxidation pathway enzyme HADHA and (ii) the FAO activity of MDM lysates. Exposure to lactate significantly increases both the HADHA-positive cells and FAO activity compared to the untreated control (Fig. 5C and D). Collectively, these findings demonstrate that host-secreted lactate during viral infection facilitates macrophage metabolic remodeling toward a metabolically uncoupled state, thereby suppressing antibacterial activity and, thus, promoting secondary bacterial infections in the airway environment.

## DISCUSSION

The mechanism by which preceding or concurrent viral infections predispose secondary bacterial infections is complex and multifaceted. Viral infections can subvert airway physiology and mucosal immunity, which results in susceptibility to bacterial attachment and failure to control bacterial replication, respectively (8). These defects in the airway environment are exaggerated in people with chronic respiratory conditions, such as CF or COPD (17, 18, 20). Macrophages, the first line of host innate immune defense, play a decisive role in the co-infection environment by orchestrating cytokine and chemokine responses. Using a specialized *in vitro* model of well-differentiated AECs, we previously reported that the antiviral response shifts AECs metabolism toward aerobic glycolysis, resulting in increased secretion of byproducts (i.e., L-lactate) in the apical milieu (23). This metabolic rearrangement also facilitates secondary bacterial infection in the airway by promoting biofilm formation (23); however, the role of the AEC metabolic rearrangement in the airway immune response was not known. In this work, we demonstrated that AEC-secreted soluble metabolites during antiviral response impair macrophage antibacterial activity (Fig. 1). We further showed that one of the secretory metabolites, L-lactate, is pivotal to this defective physiology of macrophages by increasing macrophage oxidative phosphorylation and mitochondrial fatty acid oxidation (Fig. 6).

**Fig 6:**
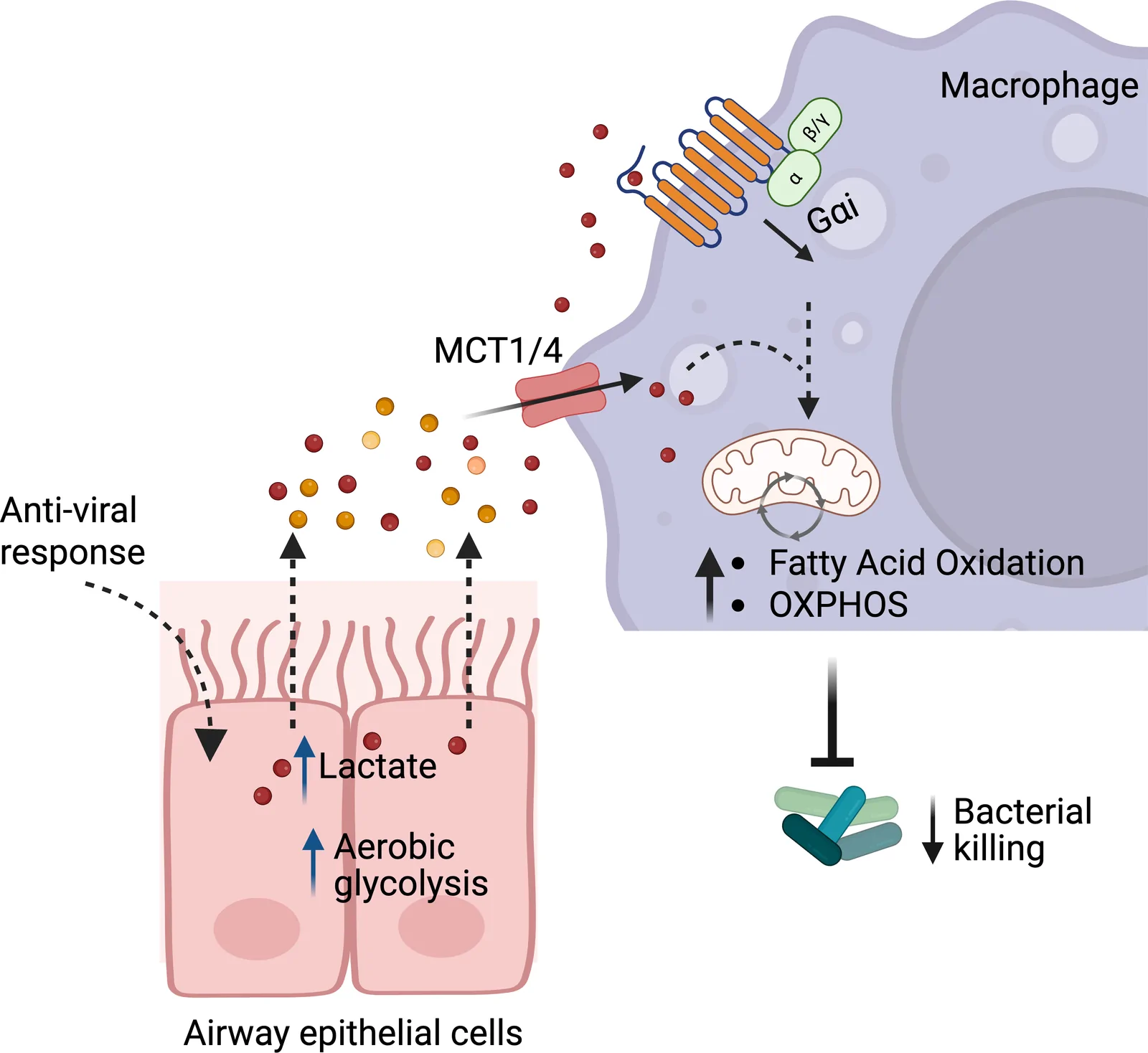
Proposed model: Airway antiviral response triggers elevated levels of lactate generation and subsequent secretion in the apical milieu, which is then transported (by MCTs) and/or sensed (by G-protein receptors) by macrophages recruited in the environment. This lactate transport/sensing facilitates macrophage toward a metabolically uncoupled state, ultimately reducing their antibacterial efficacy.

Viral infections and antiviral responses enhance host cells’ glucose metabolism through aerobic glycolysis, resembling the widely studied Warburg effect in cancer (45, 65). Aerobic glycolysis supports rapid generation of ATP to accommodate the cellular energy demand, and it also funnels metabolites to other pathways, including the pentose phosphate pathway for nucleosides, fatty acids, and antioxidant synthesis (23, 66, 67). A hallmark feature of the Warburg effect is the increased production and secretion of lactate into the extracellular environment, which has historically been considered a waste product. However, growing evidence confirms that lactate modulates immune responses in cancer (27, 68–70) and other infection environments (33, 71), suggesting that lactate could be one of the critical molecules that link metabolism and immunity. One of such examples is the muscle tissue after exercise, where tissue-resident macrophages consume lactate produced by endothelial cells and polarize into an anti-inflammatory state (72). A parallel phenomenon has been observed in the tumor microenvironment, where tumor cells secrete lactate as a means to “hijack” the surrounding macrophage population, leading to an immune shielding of the tumor (73). In addition, some studies have linked respiratory viral infections and higher levels of L-lactate with pulmonary exacerbation, including worsened bacterial infection (23, 31, 74), although the precise mechanisms are not known. Collectively, these findings have led us to more broadly consider that macrophage consumption and/or sensing of lactate may facilitate homeostatic and pro-regenerative phenotypes in co-infection environments, which inadvertently contribute to secondary bacterial infections in the airway.

Lactate typically exists as two isomers, L- and D-lactate. L-lactate is the main form found in vertebrates, and D-lactate is mainly synthesized by bacteria under anaerobic conditions (75). In a healthy physiological state, lactate (L-lactate) concentration in blood or tissues is approximately 1.5–3 mM, which can reach over 20 mM during sepsis, 40 mM in the inflamed tissues or tumor microenvironment, and ~9 mM in CF sputum (76, 77). We previously reported that, in the co-infection environment, lactate concentration increases up to 10 mM (23). Studies using a broad range of lactate concentrations demonstrated that lactate promotes macrophage anti-inflammatory polarization and suppresses proinflammatory cytokine production (i.e., TNFα and IL-6) (27, 78–80). In line with the existing literature, we also observed that 10 mM L-lactate, but not D-lactate, significantly reduces macrophage killing efficiency (Fig. 1), indicating that lactate present in the AEC secretion contributes to macrophage functional deficiencies. Moreover, this reduction in macrophage killing efficiency is a time-dependent response, with shorter incubation (60 min) with lactate having an adverse effect compared to longer incubation (24 h) (Fig. 1 and Fig. S1). This is particularly remarkable, given that incubation with a high concentration of lactate (30 mM) for shorter time durations (60 min) did not reduce macrophage killing efficiency (Fig. S2). Together, these data support that lactate production and accumulation in the host environment potentially act as a complex timer to balance macrophage pro- and anti-inflammatory responses to maintain tissue homeostasis (71, 81).

Studies have established mechanisms of lactate transport and sensing via transporters and/or receptors that affect immune cells’ pro- and anti-inflammatory phenotypes by altering cytokine production or cellular metabolic reprogramming via kinase activation and protein modification (i.e., lactylation) (34). MCT is a member of the solute carrier 16 transporter family, of which there are a total of 14 subtypes (34, 82). Among them, MCT1-4 are proton-dependent transporters involved in bidirectional transport of monocarboxylic acid (82–85). Under physiological conditions, MCT1-4 can shuttle lactate between glycolytic and oxidizing cells; however, due to its high affinity to lactate, MCT1 transports lactate according to the transmembrane gradient to maintain an intracellular and extracellular homeostasis (44, 86). Conversely, MCT4 is a hypoxia-inducible MCT with low affinity for lactate and is adapted to release lactate from tumor cells (87, 88). Such MCT-mediated lactate transport contributes to the activation or repression of transcription factor nuclear factor kappa-B (NF-κB), thereby regulating the inflammatory response (33, 44, 89). Consequently, inhibition of MCTs suppresses tumor growth and enhances immunotherapy, leading to the emergence of lactate metabolism-targeting drug development (35, 48). We, therefore, incubated macrophages with MCT inhibitor (non-selective) 2-cyano-3-(4-hydroxyphenyl)−2-propenoic acid (CHC) and examined their antibacterial activity with or without lactate exposure. Our data showed that inhibition of MCTs restores macrophage bacterial killing efficiency (Fig. 2), suggesting lactate uptake and potential intracellular accumulation are associated with the immunosuppressive effect. In addition to the transporters, two G-protein-coupled receptors (GPCRs), GPR81 and GPR132, for lactate have been reported (46, 47). GPR81 is relatively high in adipose tissue but is expressed in other cell types (80). A broad range of lactate concentrations (0.1–30 mM) can activate GPR81, suggesting the metabolite can function as an autocrine and paracrine molecule (90). GPR132 is a member of the pH-sensing GPCR family, exclusively expressed in macrophages and other hematopoietic lineages but is absent in adipocytes or cancer cells (91). GPR81 and GPR132 share amino acid homology and signal through the Gαi pathway (92). Accordingly, we incubated macrophages with Gαi pathway inhibitor pertussis toxin (PTX) and examined their antibacterial activity upon exposure to lactate. We found that inhibition of the Gαi pathway also restores macrophage bacterial killing efficiency in the presence of lactate (Fig. 2). Together, this observation suggests that both GPR-mediated lactate sensing and MCT-associated lactate transport are important to skew macrophage antibacterial activity; which is similar to the recent report by da Silva et al. that lactate uses both MCT-1 and GPR81-mediated pathways to suppress macrophage NF-κB activity (33).

The anti-inhibitory effect of lactate on LPS-stimulated macrophages has been shown previously by others (79, 93). However, all those studies have used longer incubation of macrophages and lactate. When investigating macrophage anti-inflammatory phenotypes (acknowledging the plasticity exists within the anti-inflammatory state), such as cytokines, cell markers, reactive oxygen levels, or lysosomal pH, we did not observe any significant change in these properties after 60 min of lactate incubation (Fig. 4). This observation is not atypical, given that 60-min exposure may not be enough to visualize changes in those physiological properties. A study by Shi et al. also supported this as their RNAseq analysis of bone marrow-derived macrophages (BMDMs) stimulated with LPS and lactate for 3 h showed no significant induction of anti-inflammatory marker genes. Further kinetic experiments by qPCR revealed that inhibition of proinflammatory genes by lactate occurs as early as 1 h, while the induction of anti-inflammatory genes (i.e., Arg1 and Retnla) is a later event (>3–6 h) (48). Besides these changes, pro- and anti-inflammatory macrophages are also characterized by specific metabolic adaptations to support their activities and polarization in specific contexts. Proinflammatory macrophages show increased glycolysis and accumulation of itaconate and succinate due to TCA cycle brakes, leading to hypoxia-inducible factor 1α (HIF1α) stabilization and sustained glycolytic metabolism. Conversely, anti-inflammatory macrophages rely on OXPHOS and FAO, which provide substrates for the TCA cycle and electron transport chain (55). When investigated for these immunometabolic changes, we found that lactate exposure significantly elevates macrophage OXPHOS metabolism, particularly basal respiration, and also induces FAO and expression of enzymes associated with the FAO pathway (Fig. 5). Collectively, our findings demonstrate that lactate present in the co-infection environment downregulates macrophage antimicrobial function by driving a metabolically uncoupled state (Fig. 6).

Our work highlights a new mechanism of macrophage-epithelial crosstalk during viral infection; however, we acknowledge that other metabolites in apical secretions may also contribute to macrophage function in the clinical setting. In summary, our findings highlight a mechanism behind the widespread clinical observation that viral infections predispose the acquisition of secondary bacterial infections, particularly in people with chronic lung diseases, such as CF. By better understanding the mechanistic details of the complex interactions among respiratory pathogens, host metabolites, and the immune system, we can refine the existing therapeutic targets, including the potential use of metabolic inhibitor therapeutics.

## MATERIALS AND METHODS

### Cell lines and bacterial strains

Immortalized human CF airway epithelial cell lines CFBE41o- (referred to herein as AECs) were cultured in minimal essential medium (MEM) supplemented with 10% fetal bovine serum (FBS), 2 mM L-glutamine, 5 U/mL penicillin-5 μg/mL streptomycin (P/S), and 0.5 mg/mL plasmocin prophylactic at 37°C in 5% CO_2_ (21, 23). Cells were seeded on 12-mm transwell filters (Coster) for 8–10 days at the air-liquid interface. After well polarization/differentiation, cells were basolaterally treated with 1,000 U/mL IFNβ (R&D) for 18 h at 37°C with 5% CO_2_. For bacterial strains, *Pseudomonas aeruginosa* MPAO1 (a derivative subline of PAO1), clinical isolate DH1137 (36), and fluorescent strain PAO1-tdTomato were used. Unless otherwise mentioned, bacteria were grown aerobically at 37°C in LB broth (Lennox, Millipore Sigma).

### Monocyte-derived macrophage isolation

Human monocyte-derived macrophages (MDMs) were generated from de-identified healthy donor peripheral blood mononuclear cells (PBMCs) purchased from the DartLab (Dartmouth Hitchcock Medical Center). PBMCs were isolated from blood LRS cones via a Ficoll-density gradient, and monocytes were negatively selected from PBMCs using the Classical Monocyte Isolation Kit (Miltenyi) (21). Isolated monocytes were seeded in non-tissue culture plates and cultured with 100 ng/mL recombinant human macrophage colony-stimulating factor (rhM-CSF; R&D) in cRPMI medium (RPMI-1640 supplemented with 10% FBS, 2 mM GlutaMAX-I, and 100 U/mL penicillin-100 μg/mL streptomycin) for 6–7 days to differentiate into macrophages. Twenty-four hours prior to all treatments (days 6–7 of macrophage culture), the culture medium was changed to antibiotic-free cRPMI (RPMI-1640 supplemented with 100 ng/mL rhM-CSF, 10% FBS, and 2 mM GlutaMAX-I).

### Antibiotic protection-based killing assay

Macrophage ability to clear bacterial pathogens after lactate treatment was examined using the well-established killing assay (21, 28). Macrophages were cultured in duplicate per condition in 48-well non-tissue culture plates as described above. At 60 min/24 h before the assay, macrophages were treated with 10 mM lactate or left untreated. Next, cells were challenged with bacteria (MOI = 10) and centrifuged at 500 × *g* for 5 min to promote macrophage-bacteria interaction, followed by 30-min incubation at 37°C with 5% CO_2_. Afterward, cells were incubated for 1 h with antibiotic cocktail medium (RPMI supplemented with 500 μg/mL gentamicin and 500 U/mL penicillin–500 μg/mL streptomycin) to kill extracellular bacteria. One duplicate well of cells was then washed twice with DPBS (−/−), immediately lysed with 0.1% Triton X-100, serially diluted in PBS, and spotted on LB agar plates to measure bacterial uptake CFU. The remaining duplicate well was given low-antibiotic cRPMI medium (RPMI supplemented with 10% FBS, 25 μg/mL gentamicin, and 500 U/mL penicillin-500 μg/mL streptomycin) and incubated for an additional 2 h before being lysed to collect bacterial survival CFUs. Results are calculated as percent macrophage killing efficiency {[(avg. CFU uptake − avg. CFU survival) / avg. CFU uptake] × 100}, where avg. is the average of at least two technical replicates, or calculated as percent bacteria remaining [(avg. CFU survival / avg. CFU uptake) × 100]. Percent bacteria remaining represents the bacterial CFU after 2 h post-infection (survival CFUs from the above assay) compared to the CFU at 0 min (uptake CFUs from the above assay).

### Antibiotic protection-based killing assay with apical medium from AECs

The influence of host-secreted metabolites on macrophage antibacterial activity was examined using a killing assay with apical medium. AECs were grown as mentioned earlier and basolaterally treated with 1,000 U/mL IFNβ (R&D) for 18 h at 37°C with 5% CO_2_. After 18-h incubation, the apical medium was collected and centrifuged at 3,000 × *g* for 3 min to remove cell debris. The collected supernatants were used to treat macrophages for 60 min prior to performing the killing assay. To separate extracellular vesicles, the supernatants were again ultracentrifuged at 100,000 × *g* for 60 min using Optima TLX Ultracentrifuge. The soluble fraction was used in the killing assay, and pellet (EV) was solubilized in 900 μL of RPMI and used in the killing assay. Macrophage killing efficiency was calculated as above and normalized to the IFNβ- control.

### Fluorescent PAO1 and latex-bead uptake assay

Macrophage uptake efficacy was analyzed using fluorescently labeled PAO1 or latex beads. Macrophages were incubated with 10 mM lactate for 60 min, followed by challenge with td-Tomato PAO1 (MOI = 10), or 1 μm latex beads (Sigma) (MOI = 5). Cells were incubated for 30 min at 37°C with 5% CO_2_, washed with PBS (2×), and lifted from the plate using 200 μL cell dissociation buffer (Gibco). After centrifugation for 3 min at 1,500 rpm, the cell pellet was resuspended in 200 μL pre-warmed FACS buffer (2% FBS and 1 mM EDTA in DPBS −/−) supplemented with 0.25 μg/mL propidium iodide. Cells were incubated in the dark for an additional 15 min at 37°C and 5% CO2 and then analyzed using the Beckman Coulter CytoFlex flow cytometer. At least 10,000 events were recorded, and data were analyzed using FlowJo 10.10.0 software. For microscopic visualization, macrophages were seeded on uncoated 48-well MatTek plates and visualized under the Nikon Eclipse Ti2 microscope. Representative images are shown, and the scale bar is 100 μm.

### Antibiotic protection-based killing assay using antagonists

Macrophages were incubated with monocarboxylate transporters (MCT1 and MCT4) inhibitor 2-cyano-3-(4-isopropylphenyl)−2-propenoic acid (CHC) and G-protein signaling Gαi pathway inhibitor pertussis toxin (PTX) to examine the effect of these pathways on lactate transport or sensing, respectively (27, 33, 94). Macrophages were incubated with 0.5 mM CHC (Sigma) for 1 h or with 100 ng/mL PTX (Calbiochem) for 17 h before performing the killing assay, as described above.

### Cytokine analysis

Cytokine production by macrophages was analyzed using the Luminex human 48-plex assay. Macrophages were treated with 10 mM lactate for 60 min, followed by 2 h of incubation with MPAO1. After bacterial incubation, the macrophage supernatant was collected for cytokine analysis.

### Cell surface marker expression assessment

Macrophage cell surface marker expression following lactate exposure was analyzed using a flow cytometry-based assay. Macrophages were treated with 10 mM of lactate for the mentioned time durations, washed with PBS, and lifted from the plate using cell dissociation buffer (Gibco). For extracellular markers (CD-14, CD-163, and CD-206), cells were incubated with BD staining solution supplemented with CD14-APC, CD163-PE-cyan, and CD206-eFluro450 (Invitrogen) at a concentration of 2.5 μg/mL, 5 μg/mL, and 5 μg/mL, respectively. For the intracellular marker (Arg1), cells were fixed and permeabilized with Cytofix/CytoPerm (BD Biosciences) according to the manufacturer’s instructions and stained with Arg1-eFluro450 (Invitrogen) antibody at 2 μg/mL. Finally, cells were resuspended in PBS and counted using a Beckman Coulter CytoFlex flow cytometer. FlowJo 10.10.0 software was used for data analysis.

### Oxygen consumption assay

Macrophage OXPHOS metabolism was evaluated using the fluorescent MitoXpress oxygen consumption assay (Agilent). In a non-tissue culture 96-well plate, 1× e5 cells/well were seeded and incubated as per the mentioned condition. Then, 30 or 60 min prior to the experiment, the medium was changed to clear RPMI supplemented with 10 mM lactate. Ten microliters of MitoXpress reagent was added to each well according to the manufacturer’s instructions, and time-resolved fluorescence was measured in a Synergy Neo2 plate reader at 30-s intervals for 90 min at 37°C.

### Agilent seahorse assay

Agilent Seahorse XF Cell Mito Stress Test was used to measure the kinetics of mitochondrial respiration in real-time. In Agilent cell culture microplates, 1× e5 cells/well (180 μL/well) were seeded in cRPMI medium as mentioned above. Then 30 or 60 min prior to the experiment, the medium was changed to clear RPMI supplemented with 10 mM lactate. The OCR of macrophages was measured on the Seahorse XFe96 (Agilent) with a final concentration of 1.5 μM oligomycin, 0.5 μM FCCP, and 0.5 μM antimycin A, according to the kit instructions. Reading was recorded for a total of 80 min, with 20 min for each section.

### Assessment of ROS production and lysosomal pH

Macrophage ROS generation and lysosomal pH gradient were evaluated using the fluorescent probes 5-(and-6)chloromethyl-2,7-dichlorohydrofluorescein diacetate acetyl ester (CM-H2DCFDA) and Lysotracker-DeepRed, respectively (Invitrogen). Untreated or 10 mM lactate-treated macrophages were challenged with MPAO1 (MOI = 10) for 30 min, lifted from plates using cell dissociation buffer (Gibco), and incubated in FACS buffer supplemented with 5 μM CM-H2DCFDA for 15 min or 50 nM Lysotracker-DeepRed for 30 min. Fluorescent signals were assessed using the Beckman Coulter CytoFlex flow cytometer, and data were analyzed with FlowJo software.

### Fatty acid oxidation assay

A flow cytometry-based and a spectrometry-based assay were used to analyze macrophage fatty acid oxidation efficiency after lactate exposure. Flow cytometry assay (Abcam) was used to examine the abundance of mitochondrial fatty acid β-oxidation pathway enzyme HADHA. Lactate-treated cells were lifted using cell dissociation buffer and fixed with 4% paraformaldehyde. Next, cells were permeabilized and stained with anti-HADHA primary and goat α-mouse IgG secondary antibody according to the kit’s instructions. Finally, cells were analyzed using a Beckman CytoFlex flow cytometer, and data were interpreted using FlowJo software. The colorimetric assay (AssayGiene) was used to quantitatively measure FAO in macrophages. Cells were isolated after lactate treatment, lysed, and the soluble protein fraction was collected. The BCA (Sigma) assay was used to quantify the protein level, and an equal amount of protein across samples was used to quantify FAO activity as per the manufacturer’s protocol.

### Statistical analysis

Each experiment was performed using at least three randomly selected deidentified donors’ PBMCs. Data are presented as mean ± SD. A linear mixed-effects model was performed in RStudio, with donor as a random effect and treatment as a fixed effect.

## SUPPLEMENTAL MATERIAL

Supplemental figures iai.00409-26-s0001.pdf Fig. S1 to S5.

ASM does not own the copyrights to Supplemental Material that may be linked to, or accessed through, an article. The authors have granted ASM a non-exclusive, world-wide license to publish the Supplemental Material files. Please contact the corresponding author directly for reuse.

## References

[1] Mathew S, Smatti MK, Al Ansari K, Nasrallah GK, Al Thani AA, Yassine HM. 2019. Mixed viral-bacterial infections and their effects on gut microbiota and clinical illnesses in children. Sci Rep 9:865. doi:10.1038/s41598-018-37162-w PMC6351549 30696865

[2] Hendricks MR, Lashua LP, Fischer DK, Flitter BA, Eichinger KM, Durbin JE, Sarkar SN, Coyne CB, Empey KM, Bomberger JM. 2016. Respiratory syncytial virus infection enhances *Pseudomonas aeruginosa* biofilm growth through dysregulation of nutritional immunity. Proc Natl Acad Sci USA 113:1642–1647. doi:10.1073/pnas.1516979113 PMC4760822 26729873

[3] Beadling C, Slifka MK. 2004. How do viral infections predispose patients to bacterial infections? Curr Opin Infect Dis 17:185–191. doi:10.1097/00001432-200406000-00003 15166819

[4] Peltola VT, McCullers JA, Fink RJ, Fedson DS. 2004. Respiratory viruses predisposing to bacterial infections: role of neuraminidase. Pediatr Infect Dis J 23:S87–97. doi:10.1097/01.inf.0000108197.81270.35 14730275

[5] Lane S, Hilliam Y, Bomberger JM. 2023. Microbial and immune regulation of the gut-lung axis during viral-bacterial coinfection. J Bacteriol 205:e0029522. doi:10.1128/jb.00295-22 PMC9879096 36409130

[6] Wang EE, Prober CG, Manson B, Corey M, Levison H. 1984. Association of respiratory viral infections with pulmonary deterioration in patients with cystic fibrosis. N Engl J Med 311:1653–1658. doi:10.1056/NEJM198412273112602 6504106

[7] Melvin JA, Lashua LP, Kiedrowski MR, Yang G, Deslouches B, Montelaro RC, Bomberger JM. 2016. Simultaneous antibiofilm and antiviral activities of an engineered antimicrobial peptide during virus-bacterium coinfection. mSphere 1:e00083-16. doi:10.1128/mSphere.00083-16 PMC4888888 27303744

[8] Bakaletz LO. 2017. Viral-bacterial co-infections in the respiratory tract. Curr Opin Microbiol 35:30–35. doi:10.1016/j.mib.2016.11.003 PMC7108227 27940028

[9] Manna S, McAuley J, Jacobson J, Nguyen CD, Ullah MdA, Sebina I, Williamson V, Mulholland EK, Wijburg O, Phipps S, Satzke C. 2022. Synergism and antagonism of bacterial-viral coinfection in the upper respiratory tract. mSphere 7:e00984–21. doi:10.1128/msphere.00984-21 PMC8769199 35044807

[10] Barr R, Green CA, Sande CJ, Drysdale SB. 2019. Respiratory syncytial virus: diagnosis, prevention and management. Ther Adv Infect Dis 6:2049936119865798. doi:10.1177/2049936119865798 PMC6664627 31384456

[11] Kiedrowski MR, Bomberger JM. 2018. Viral-bacterial co-infections in the cystic fibrosis respiratory tract. Front Immunol 9:3067. doi:10.3389/fimmu.2018.03067 PMC6306490 30619379

[12] Italiani P, Boraschi D. 2014. From monocytes to M1/M2 macrophages: phenotypical vs. functional differentiation. Front Immunol 5:1–22. doi:10.3389/fimmu.2014.00514 PMC4201108 25368618

[13] Byrne AJ, Mathie SA, Gregory LG, Lloyd CM. 2015. Pulmonary macrophages: key players in the innate defence of the airways. Thorax 70:1189–1196. doi:10.1136/thoraxjnl-2015-207020 26286722

[14] Marrufo AM, Flores-Mireles AL. 2024. Macrophage fate: to kill or not to kill? Infect Immun 92:e0047623. doi:10.1128/iai.00476-23 PMC11385966 38829045

[15] Chen S, Saeed AFUH, Liu Q, Jiang Q, Xu H, Xiao GG, Rao L, Duo Y. 2023. Macrophages in immunoregulation and therapeutics. Signal Transduct Target Ther 8:207. doi:10.1038/s41392-023-01452-1 PMC10200802 37211559

[16] Chen S, Yang J, Wei Y, Wei X. 2020. Epigenetic regulation of macrophages: from homeostasis maintenance to host defense. Cell Mol Immunol 17:36–49. doi:10.1038/s41423-019-0315-0 PMC6952359 31664225

[17] Bain CC, MacDonald AS. 2022. The impact of the lung environment on macrophage development, activation and function: diversity in the face of adversity. Mucosal Immunol 15:223–234. doi:10.1038/s41385-021-00480-w PMC8749355 35017701

[18] Turton KB, Ingram RJ, Valvano MA. 2021. Macrophage dysfunction in cystic fibrosis: nature or nurture? J Leukoc Biol 109:573–582. doi:10.1002/JLB.4RU0620-245R 32678926

[19] Schupp JC, Khanal S, Gomez JL, Sauler M, Adams TS, Chupp GL, Yan X, Poli S, Zhao Y, Montgomery RR, Rosas IO, Dela Cruz CS, Bruscia EM, Egan ME, Kaminski N, Britto CJ. 2020. Single-cell transcriptional archetypes of airway inflammation in cystic fibrosis. Am J Respir Crit Care Med 202:1419–1429. doi:10.1164/rccm.202004-0991OC PMC7667912 32603604

[20] Hoffmann J, Machado D, Terrier O, Pouzol S, Messaoudi M, Basualdo W, Espínola EE, Guillen RM, Rosa-Calatrava M, Picot V, Bénet T, Endtz H, Russomando G, Paranhos-Baccalà G. 2016. Viral and bacterial co-infection in severe pneumonia triggers innate immune responses and specifically enhances IP-10: a translational study. Sci Rep 6:38532. doi:10.1038/srep38532 PMC5138590 27922126

[21] Lane S, White TLA, Walsh EE, Cattley RT, Cumberland R, Hawse WF, Delgoffe GM, Badylak SF, Bomberger JM. 2023. Antiviral epithelial-macrophage crosstalk permits secondary bacterial infections. mBio 14:e0086323. doi:10.1128/mbio.00863-23 PMC10653878 37772820

[22] Lévêque M, Le Trionnaire S, Del Porto P, Martin-Chouly C. 2017. The impact of impaired macrophage functions in cystic fibrosis disease progression. J Cyst Fibros 16:443–453. doi:10.1016/j.jcf.2016.10.011 27856165

[23] Carreno-Florez GP, Kocak BR, Hendricks MR, Melvin JA, Mar KB, Kosanovich J, Cumberland RL, Delgoffe GM, Shiva S, Empey KM, Schoggins JW, Bomberger JM. 2023. Interferon signaling drives epithelial metabolic reprogramming to promote secondary bacterial infection. PLoS Pathog 19:e1011719. doi:10.1371/journal.ppat.1011719 PMC10631704 37939149

[24] Levin L, Gevers W. 1981. Metabolic alterations in cancer. Part I. Carbohydrate metabolism. S Afr Med J 59:518–521. 7013105

[25] Wang ZH, Peng WB, Zhang P, Yang XP, Zhou Q. 2021. Lactate in the tumour microenvironment: from immune modulation to therapy. EBioMedicine 73:103627. doi:10.1016/j.ebiom.2021.103627 PMC8524104 34656878

[26] Llibre A, Kucuk S, Gope A, Certo M, Mauro C. 2025. Lactate: a key regulator of the immune response. Immunity 58:535–554. doi:10.1016/j.immuni.2025.02.008 40073846

[27] Colegio OR, Chu N-Q, Szabo AL, Chu T, Rhebergen AM, Jairam V, Cyrus N, Brokowski CE, Eisenbarth SC, Phillips GM, Cline GW, Phillips AJ, Medzhitov R. 2014. Functional polarization of tumour-associated macrophages by tumour-derived lactic acid. Nature 513:559–563. doi:10.1038/nature13490 PMC4301845 25043024

[28] Florek LC, Lin X, Lin Y-C, Lin M-H, Chakraborty A, Price-Whelan A, Tong L, Rahme L, Dietrich LEP. 2024. The L-lactate dehydrogenases of *Pseudomonas aeruginosa* are conditionally regulated but both contribute to survival during macrophage infection. bioRxiv:2024.03.21.586142. doi:10.1101/2024.03.21.586142 PMC11389411 39162563

[29] Palmer CS. 2022. Innate metabolic responses against viral infections. Nat Metab 4:1245–1259. doi:10.1038/s42255-022-00652-3 36266542

[30] Ren L, Zhang W, Zhang J, Zhang J, Zhang H, Zhu Y, Meng X, Yi Z, Wang R. 2021. Influenza A virus (H1N1) infection induces glycolysis to facilitate viral replication. Virol Sin 36:1532–1542. doi:10.1007/s12250-021-00433-4 PMC8692537 34519916

[31] Bensel T, Stotz M, Borneff-Lipp M, Wollschläger B, Wienke A, Taccetti G, Campana S, Meyer KC, Jensen PØ, Lechner U, Ulrich M, Döring G, Worlitzsch D. 2011. Lactate in cystic fibrosis sputum. J Cyst Fibros 10:37–44. doi:10.1016/j.jcf.2010.09.004 20947455

[32] de la Paz Gutierrez M, Hall JL, Winter SE, Rivera-Chávez F. 2025. Cholera toxin-induced disease generates epithelial cell-derived L-lactate that promotes *Vibrio cholerae* growth in the small intestine. Microbiology. doi:10.1101/2025.08.12.669941 PMC13528590 42262925

[33] Silva RAG, Yin B, Tien Q, Hsien P, Kao N, Thibault G, et al.. 2026. *Enterococcus faecalis* -derived lactic acid suppresses macrophage activation to facilitate persistent and polymicrobial wound infections *Enterococcus faecalis* -derived lactic acid suppresses macrophage activation to facilitate persistent and polymi. Cell Host Microbe:1–18. doi:10.1016/j.chom.2026.01.002 41605216

[34] Certo M, Llibre A, Lee W, Mauro C. 2022. Understanding lactate sensing and signalling. Trends Endocrinol Metab 33:722–735. doi:10.1016/j.tem.2022.07.004 35999109

[35] Chen J, Huang Z, Chen Y, Tian H, Chai P, Shen Y, Yao Y, Xu S, Ge S, Jia R. 2025. Lactate and lactylation in cancer. Signal Transduct Target Ther 10:38. doi:10.1038/s41392-024-02082-x PMC11814237 39934144

[36] Smith EE, Buckley DG, Wu Z, Saenphimmachak C, Hoffman LR, D’Argenio DA, Miller SI, Ramsey BW, Speert DP, Moskowitz SM, Burns JL, Kaul R, Olson MV. 2006. Genetic adaptation by *Pseudomonas aeruginosa* to the airways of cystic fibrosis patients. Proc Natl Acad Sci USA 103:8487–8492. doi:10.1073/pnas.0602138103 PMC1482519 16687478

[37] Li X, Yang Y, Zhang B, Lin X, Fu X, An Y, Zou Y, Wang J-X, Wang Z, Yu T. 2022. Lactate metabolism in human health and disease. Sig Transduct Target Ther 7:36050306. doi:10.1038/s41392-022-01151-3 PMC9434547 36050306

[38] Heim C, Bosch ME, Yamada KJ, Aldrich AL, Chaudhari SS, Klinkebiel D, Gries CM, Alqarzaee AA, Li Y, Thomas VC, Seto E, Kielian T. 2021. Lactate production by *Staphylococcus aureus* biofilm inhibits HDAC11 to reprogram the host immune response during persistent infection. J Immunol 206:110. doi:10.4049/jimmunol.206.Supp.110.09 PMC7529909 32661313

[39] Walenta S, Wetterling M, Lehrke M, Schwickert G, Sundfør K, Rofstad EK, Mueller-Klieser W. 2000. High lactate levels predict likelihood of metastases, tumor recurrence, and restricted patient survival in human cervical cancers. Cancer Res 60:916–921. 10706105

[40] O’Callaghan AA, Dempsey E, Iyer N, Stiegeler S, Mercurio K, Corr SC. 2021. Intestinal metabolites influence macrophage phagocytosis and clearance of bacterial infection. Front Cell Infect Microbiol 11:622491. doi:10.3389/fcimb.2021.622491 PMC8327167 34350128

[41] Weißelberg S, Both A, Failla AV, Huang J, Linder S, Ohnezeit D, Bartsch P, Aepfelbacher M, Rohde H. 2024. *Staphylococcus epidermidis* alters macrophage polarization and phagocytic uptake by extracellular DNA release *in vitro*. NPJ Biofilms Microbiomes 10:131. doi:10.1038/s41522-024-00604-7 PMC11579364 39567551

[42] Tomlinson KL, Prince AS, Wong Fok Lung T. 2021. Immunometabolites drive bacterial adaptation to the airway. Front Immunol 12:790574. doi:10.3389/fimmu.2021.790574 PMC8656696 34899759

[43] Machado MG, Sencio V, Trottein F. 2021. Short-chain fatty acids as a potential treatment for infections: a closer look at the lungs. Infect Immun 89:e0018821. doi:10.1128/IAI.00188-21 PMC8370681 34097474

[44] Fang Y, Li Z, Yang L, Li W, Wang Y, Kong Z, Miao J, Chen Y, Bian Y, Zeng L. 2024. Emerging roles of lactate in acute and chronic inflammation. Cell Commun Signal 22:276. doi:10.1186/s12964-024-01624-8 PMC11097486 38755659

[45] San-Millán I, Brooks GA. 2017. Reexamining cancer metabolism: lactate production for carcinogenesis could be the purpose and explanation of the Warburg Effect. Carcinogenesis 38:119–133. doi:10.1093/carcin/bgw127 PMC5862360 27993896

[46] Roland CL, Arumugam T, Deng D, Liu SH, Philip B, Gomez S, Burns WR, Ramachandran V, Wang H, Cruz-Monserrate Z, Logsdon CD. 2014. Cell surface lactate receptor GPR81 is crucial for cancer cell survival. Cancer Res 74:5301–5310. doi:10.1158/0008-5472.CAN-14-0319 PMC4167222 24928781

[47] Chen P, Zuo H, Xiong H, Kolar MJ, Chu Q, Saghatelian A, Siegwart DJ, Wan Y. 2017. Gpr132 sensing of lactate mediates tumor-macrophage interplay to promote breast cancer metastasis. Proc Natl Acad Sci USA 114:580–585. doi:10.1073/pnas.1614035114 PMC5255630 28049847

[48] Shi W, Cassmann TJ, Bhagwate AV, Hitosugi T, Ip WKE. 2024. Lactic acid induces transcriptional repression of macrophage inflammatory response via histone acetylation. Cell Rep 43:113746. doi:10.1016/j.celrep.2024.113746 PMC10957222 38329873

[49] Keen AC, Pedersen MH, Lemel L, Scott DJ, Canals M, Littler DR, Beddoe T, Ono Y, Shi L, Inoue A, Javitch JA, Lane JR. 2022. OZITX, a pertussis toxin-like protein for occluding inhibitory G protein signalling including Gα_z_ Commun Biol 5:256. doi:10.1038/s42003-022-03191-5 PMC8943041 35322196

[50] Lattin J, Zidar DA, Schroder K, Kellie S, Hume DA, Sweet MJ. 2007. G-protein-coupled receptor expression, function, and signaling in macrophages. J Leukoc Biol 82:16–32. doi:10.1189/jlb.0107051 17456803

[51] Hoggatt J, Singh P, Tate TA, Chou B-K, Datari SR, Fukuda S, Liu L, Kharchenko PV, Schajnovitz A, Baryawno N, Mercier FE, Boyer J, Gardner J, Morrow DM, Scadden DT, Pelus LM. 2018. Rapid mobilization reveals a highly engraftable hematopoietic stem cell. Cell 172:191–204. doi:10.1016/j.cell.2017.11.003 PMC5812290 29224778

[52] Batchelor RH, Zhou M. 2004. Use of cellular glucose-6-phosphate dehydrogenase for cell quantitation: applications in cytotoxicity and apoptosis assays. Anal Biochem 329:35–42. doi:10.1016/j.ab.2004.02.007 15136165

[53] Takiguchi H, Yang CX, Yang CWT, Sahin B, Whalen BA, Milne S, Akata K, Yamasaki K, Yang JSW, Cheung CY, Vander Werff R, McNagny KM, Leitao Filho FS, Shaipanich T, van Eeden SF, Obeidat M, Leung JM, Sin DD. 2021. Macrophages with reduced expressions of classical M1 and M2 surface markers in human bronchoalveolar lavage fluid exhibit pro-inflammatory gene signatures. Sci Rep 11:1–11. doi:10.1038/s41598-021-87720-y PMC8050093 33859282

[54] Parisi L, Gini E, Baci D, Tremolati M, Fanuli M, Bassani B, Farronato G, Bruno A, Mortara L. 2018. Macrophage polarization in chronic inflammatory diseases: killers or builders? J Immunol Res 2018:8917804. doi:10.1155/2018/8917804 PMC5821995 29507865

[55] Viola A, Munari F, Sánchez-Rodríguez R, Scolaro T, Castegna A. 2019. The metabolic signature of macrophage responses. Front Immunol 10. doi:10.3389/fimmu.2019.01462 PMC6618143 31333642

[56] Geng J, Sun X, Wang P, Zhang S, Wang X, Wu H, Hong L, Xie C, Li X, Zhao H, Liu Q, Jiang M, Chen Q, Zhang J, Li Y, Song S, Wang H-R, Zhou R, Johnson RL, Chien K-Y, Lin S-C, Han J, Avruch J, Chen L, Zhou D. 2015. Kinases Mst1 and Mst2 positively regulate phagocytic induction of reactive oxygen species and bactericidal activity. Nat Immunol 16:1142–1152. doi:10.1038/ni.3268 PMC4618176 26414765

[57] Lemaire S, Mingeot-Leclercq M-P, Tulkens PM, Van Bambeke F. 2014. Study of macrophage functions in murine J774 cells and human activated THP-1 cells exposed to oritavancin, a lipoglycopeptide with high cellular accumulation. Antimicrob Agents Chemother 58:2059–2066. doi:10.1128/AAC.02475-13 PMC4023769 24449768

[58] Geeraerts X, Fernández-Garcia J, Hartmann FJ, de Goede KE, Martens L, Elkrim Y, Debraekeleer A, Stijlemans B, Vandekeere A, Rinaldi G, De Rycke R, Planque M, Broekaert D, Meinster E, Clappaert E, Bardet P, Murgaski A, Gysemans C, Nana FA, Saeys Y, Bendall SC, Laoui D, Van den Bossche J, Fendt S-M, Van Ginderachter JA. 2021. Macrophages are metabolically heterogeneous within the tumor microenvironment. Cell Rep 37:110171. doi:10.1016/j.celrep.2021.110171 34965415

[59] Wculek SK, Dunphy G, Heras-Murillo I, Mastrangelo A, Sancho D. 2022. Metabolism of tissue macrophages in homeostasis and pathology. Cell Mol Immunol 19:384–408. doi:10.1038/s41423-021-00791-9 PMC8891297 34876704

[60] Galli G, Saleh M. 2020. Immunometabolism of macrophages in bacterial infections. Front Cell Infect Microbiol 10:607650. doi:10.3389/fcimb.2020.607650 PMC7879570 33585278

[61] Galván-Peña S, O’Neill LAJ. 2014. Metabolic reprograming in macrophage polarization. Front Immunol 5:420. doi:10.3389/fimmu.2014.00420 PMC4151090 25228902

[62] Chandra P, He L, Zimmerman M, Yang G, Köster S, Ouimet M, Wang H, Moore KJ, Dartois V, Schilling JD, Philips JA. 2020. Inhibition of fatty acid oxidation promotes macrophage control of *Mycobacterium tuberculosis*. mBio 11:1–15. doi:10.1128/mBio.01139-20 PMC7343992 32636249

[63] Sankar P, Ramos RB, Corro J, Mishra LK, Nafiz TN, Bhargavi G, Saqib M, Poswayo SKL, Parihar SP, Cai Y, Subbian S, Ojha AK, Mishra BB. 2024. Fatty acid metabolism in neutrophils promotes lung damage and bacterial replication during tuberculosis. PLoS Pathog 20:e1012188. doi:10.1371/journal.ppat.1012188 PMC11482725 39365825

[64] Zhang D, Tang Z, Huang H, Zhou G, Cui C, Weng Y, Liu W, Kim S, Lee S, Perez-Neut M, Ding J, Czyz D, Hu R, Ye Z, He M, Zheng YG, Shuman HA, Dai L, Ren B, Roeder RG, Becker L, Zhao Y. 2019. Metabolic regulation of gene expression by histone lactylation. Nature 574:575–580. doi:10.1038/s41586-019-1678-1 PMC6818755 31645732

[65] WARBURG O. 1956. On the origin of cancer cells. Science 123:309–314. doi:10.1126/science.123.3191.309 13298683

[66] Lunt SY, Vander Heiden MG. 2011. Aerobic glycolysis: meeting the metabolic requirements of cell proliferation. Annu Rev Cell Dev Biol 27:441–464. doi:10.1146/annurev-cellbio-092910-154237 21985671

[67] Schulze A, Harris AL. 2012. How cancer metabolism is tuned for proliferation and vulnerable to disruption. Nature 491:364–373. doi:10.1038/nature11706 23151579

[68] Yang K, Xu J, Fan M, Tu F, Wang X, Ha T, Williams DL, Li C. 2020. Lactate suppresses macrophage pro-inflammatory response to LPS stimulation by inhibition of YAP and NF-κB activation *via* GPR81-mediated signaling. Front Immunol 11:587913. doi:10.3389/fimmu.2020.587913 PMC7573489 33123172

[69] de la Cruz-López KG, Castro-Muñoz LJ, Reyes-Hernández DO, García-Carrancá A, Manzo-Merino J. 2019. Lactate in the regulation of tumor microenvironment and therapeutic approaches. Front Oncol 9:9–2019. doi:10.3389/fonc.2019.01143 PMC6839026 31737570

[70] Brand A, Singer K, Koehl GE, Kolitzus M, Schoenhammer G, Thiel A, Matos C, Bruss C, Klobuch S, Peter K, et al.. 2016. LDHA-associated lactic acid production blunts tumor immunosurveillance by T and NK cells. Cell Metab 24:657–671. doi:10.1016/j.cmet.2016.08.011 27641098

[71] Wang X, Yang B, Ma S, Yan X, Ma S, Sun H, Sun Y, Jiang L. 2023. Lactate promotes *Salmonella* intracellular replication and systemic infection via driving macrophage M2 polarization. Microbiol Spectr 11. doi:10.1128/spectrum.02253-23 PMC10715217 37796020

[72] Zhang J, Muri J, Fitzgerald G, Gorski T, Gianni-Barrera R, Masschelein E, D’Hulst G, Gilardoni P, Turiel G, Fan Z, Wang T, Planque M, Carmeliet P, Pellerin L, Wolfrum C, Fendt S-M, Banfi A, Stockmann C, Soro-Arnáiz I, Kopf M, De Bock K. 2020. Endothelial lactate controls muscle regeneration from ischemia by inducing M2-like macrophage polarization. Cell Metab 31:1136–1153. doi:10.1016/j.cmet.2020.05.004 PMC7267778 32492393

[73] Li Z, Wang Q, Huang X, Yang M, Zhou S, Li Z, Fang Z, Tang Y, Chen Q, Hou H, Li L, Fei F, Wang Q, Wu Y, Gong A. 2023. Lactate in the tumor microenvironment: a rising star for targeted tumor therapy. Front Nutr 10. doi:10.3389/fnut.2023.1113739 PMC9978120 36875841

[74] Fredman G, Kolpen M, Hertz FB, Petersen PT, Jensen AV, Baunbaek-Egelund G, Ravn P, Jensen PØ, Faurholt-Jepsen D. 2019. The inflamed sputum in lower respiratory tract infection: l-lactate levels are correlated to neutrophil accumulation. APMIS 127:72–79. doi:10.1111/apm.12913 PMC7159756 30614067

[75] Pohanka M. 2020. D‐lactic acid as a metabolite: toxicology, diagnosis, and detection. Edited by M. P. Evstigneev. Biomed Res Int 2020:3419034. doi:10.1155/2020/3419034 PMC7320276 32685468

[76] Pucino V, Certo M, Bulusu V, Cucchi D, Goldmann K, Pontarini E, Haas R, Smith J, Headland SE, Blighe K, Ruscica M, Humby F, Lewis MJ, Kamphorst JJ, Bombardieri M, Pitzalis C, Mauro C. 2019. Lactate buildup at the site of chronic inflammation promotes disease by inducing CD4^+^ T cell metabolic rewiring. Cell Metab 30:1055–1074. doi:10.1016/j.cmet.2019.10.004 PMC6899510 31708446

[77] Liu Z, Meng Z, Li Y, Zhao J, Wu S, Gou S, Wu H. 2019. Prognostic accuracy of the serum lactate level, the SOFA score and the qSOFA score for mortality among adults with Sepsis. Scand J Trauma Resusc Emerg Med 27:51. doi:10.1186/s13049-019-0609-3 PMC6492372 31039813

[78] Selleri S, Bifsha P, Civini S, Pacelli C, Dieng MM, Lemieux W, Jin P, Bazin R, Patey N, Marincola FM, Moldovan F, Zaouter C, Trudeau L-E, Benabdhalla B, Louis I, Beauséjour C, Stroncek D, Le Deist F, Haddad E. 2016. Human mesenchymal stromal cell-secreted lactate induces M2-macrophage differentiation by metabolic reprogramming. Oncotarget 7:30193–30210. doi:10.18632/oncotarget.8623 PMC5058674 27070086

[79] Dietl K, Renner K, Dettmer K, Timischl B, Eberhart K, Dorn C, Hellerbrand C, Kastenberger M, Kunz-Schughart LA, Oefner PJ, Andreesen R, Gottfried E, Kreutz MP. 2010. Lactic acid and acidification inhibit TNF secretion and glycolysis of human monocytes. J Immunol 184:1200–1209. doi:10.4049/jimmunol.0902584 20026743

[80] Errea A, Cayet D, Marchetti P, Tang C, Kluza J, Offermanns S, Sirard J-C, Rumbo M. 2016. Lactate inhibits the pro-inflammatory response and metabolic reprogramming in murine macrophages in a GPR81-independent manner. Edited by J. Bayry. Plos One 11:e0163694. doi:10.1371/journal.pone.0163694 PMC5112849 27846210

[81] Rosenberg G, Riquelme S, Prince A, Avraham R. 2022. Immunometabolic crosstalk during bacterial infection. Nat Microbiol 7:497–507. doi:10.1038/s41564-022-01080-5 35365784

[82] Halestrap AP. 2012. The monocarboxylate transporter family--structure and functional characterization. IUBMB Life 64:1–9. doi:10.1002/iub.573 22131303

[83] Felmlee MA, Jones RS, Rodriguez-Cruz V, Follman KE, Morris ME. 2020. Monocarboxylate transporters (SLC16): function, regulation, and role in health and disease. Pharmacol Rev 72:466–485. doi:10.1124/pr.119.018762 PMC7062045 32144120

[84] Braga M, Kaliszczak M, Carroll L, Schug ZT, Heinzmann K, Baxan N, Benito A, Valbuena GN, Stribbling S, Beckley A, Mackay G, Mauri F, Latigo J, Barnes C, Keun H, Gottlieb E, Aboagye EO. 2020. Tracing nutrient flux following monocarboxylate transporter-1 inhibition with AZD3965. Cancers (Basel) 12:1703. doi:10.3390/cancers12061703 PMC7352845 32604836

[85] Sriram R, Van Criekinge M, Hansen A, Wang ZJ, Vigneron DB, Wilson DM, Keshari KR, Kurhanewicz J. 2015. Real-time measurement of hyperpolarized lactate production and efflux as a biomarker of tumor aggressiveness in an MR compatible 3D cell culture bioreactor. NMR Biomed 28:1141–1149. doi:10.1002/nbm.3354 PMC4537810 26202449

[86] Halestrap AP. 2013. The SLC16 gene family - structure, role and regulation in health and disease. Mol Aspects Med 34:337–349. doi:10.1016/j.mam.2012.05.003 23506875

[87] Draoui N, Feron O. 2011. Lactate shuttles at a glance: from physiological paradigms to anti-cancer treatments. Dis Model Mech 4:727–732. doi:10.1242/dmm.007724 PMC3209642 22065843

[88] Fiaschi T, Marini A, Giannoni E, Taddei ML, Gandellini P, De Donatis A, Lanciotti M, Serni S, Cirri P, Chiarugi P. 2012. Reciprocal metabolic reprogramming through lactate shuttle coordinately influences tumor-stroma interplay. Cancer Res 72:5130–5140. doi:10.1158/0008-5472.CAN-12-1949 22850421

[89] Zhang S, Xu W, Wang H, Cao M, Li M, Zhao J, Hu Y, Wang Y, Li S, Xie Y, Chen G, Liu R, Cheng Y, Xu Z, Zou K, Gong S, Geng L. 2019. Inhibition of CREB‐mediated ZO‐1 and activation of NF‐κB‐induced IL‐6 by colonic epithelial MCT4 destroys intestinal barrier function. Cell Prolif 52:e12673. doi:10.1111/cpr.12673 PMC6869122 31418947

[90] Ahmed K, Tunaru S, Tang C, Müller M, Gille A, Sassmann A, Hanson J, Offermanns S. 2010. An autocrine lactate loop mediates insulin-dependent inhibition of lipolysis through GPR81. Cell Metab 11:311–319. doi:10.1016/j.cmet.2010.02.012 20374963

[91] Justus CR, Dong L, Yang LV. 2013. Acidic tumor microenvironment and pH-sensing G protein-coupled receptors. Front Physiol 4:354. doi:10.3389/fphys.2013.00354 PMC3851830 24367336

[92] Manoharan I, Prasad PD, Thangaraju M, Manicassamy S. 2021. Lactate-dependent regulation of immune responses by dendritic cells and macrophages. Front Immunol 12:691134. doi:10.3389/fimmu.2021.691134 PMC8358770 34394085

[93] Peter K, Rehli M, Singer K, Renner-Sattler K, Kreutz M. 2015. Lactic acid delays the inflammatory response of human monocytes. Biochem Biophys Res Commun 457:412–418. doi:10.1016/j.bbrc.2015.01.005 25582773

[94] Lattin JE, Schroder K, Su AI, Walker JR, Zhang J, Wiltshire T, Saijo K, Glass CK, Hume DA, Kellie S, Sweet MJ. 2008. Expression analysis of G protein-coupled receptors in mouse macrophages. Immunome Res 4:1–13. doi:10.1186/1745-7580-4-5 PMC2394514 18442421

