# Supplementary material for: Host-secreted lactate during respiratory viral infection diminishes macrophage antibacterial activity through metabolic reprogramming
Source: Infect Immun. 2026 Oct 12;94(11):e00409-26. doi: 10.1128/iai.00409-26 (PMC13750119; doi:10.1128/iai.00409-26)
Supplement: Supplementary file 1 [file iai.00409-26-s0001.pdf]

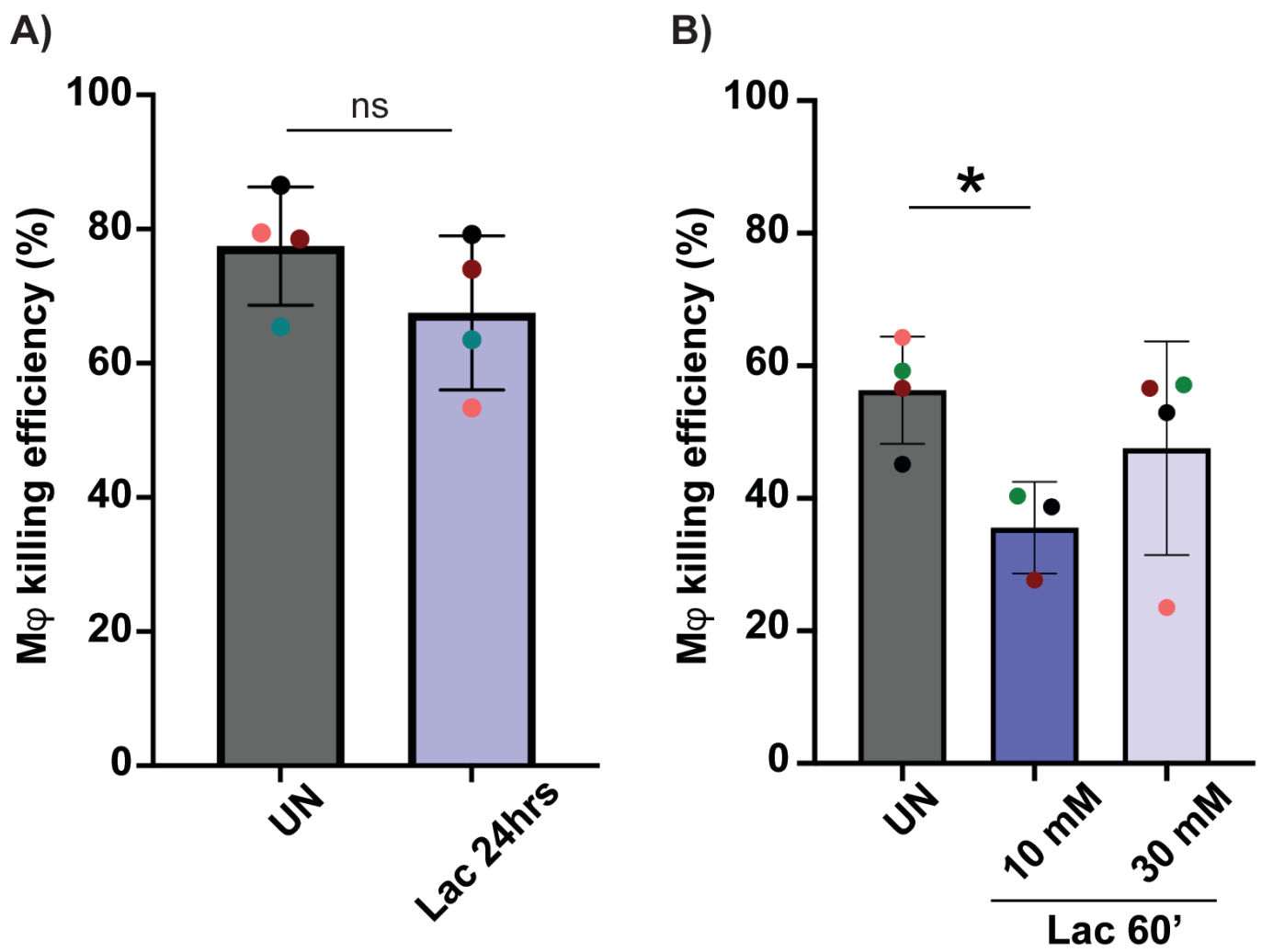

Figure S1: (A) Mφ were exposed to 10 mM lactate for 24 hr, and killing efficiency was analyzed using the killing assay. (B) Mφ were treated with 10 mM or 30 mM lactate for 60 min, followed by the killing assay to analyze macrophage killing efficiency. Each color denotes a donor. Statistics: linear mixed effects model, donor as a random effect, and treatment as a fixed effect. **p< 0.01.

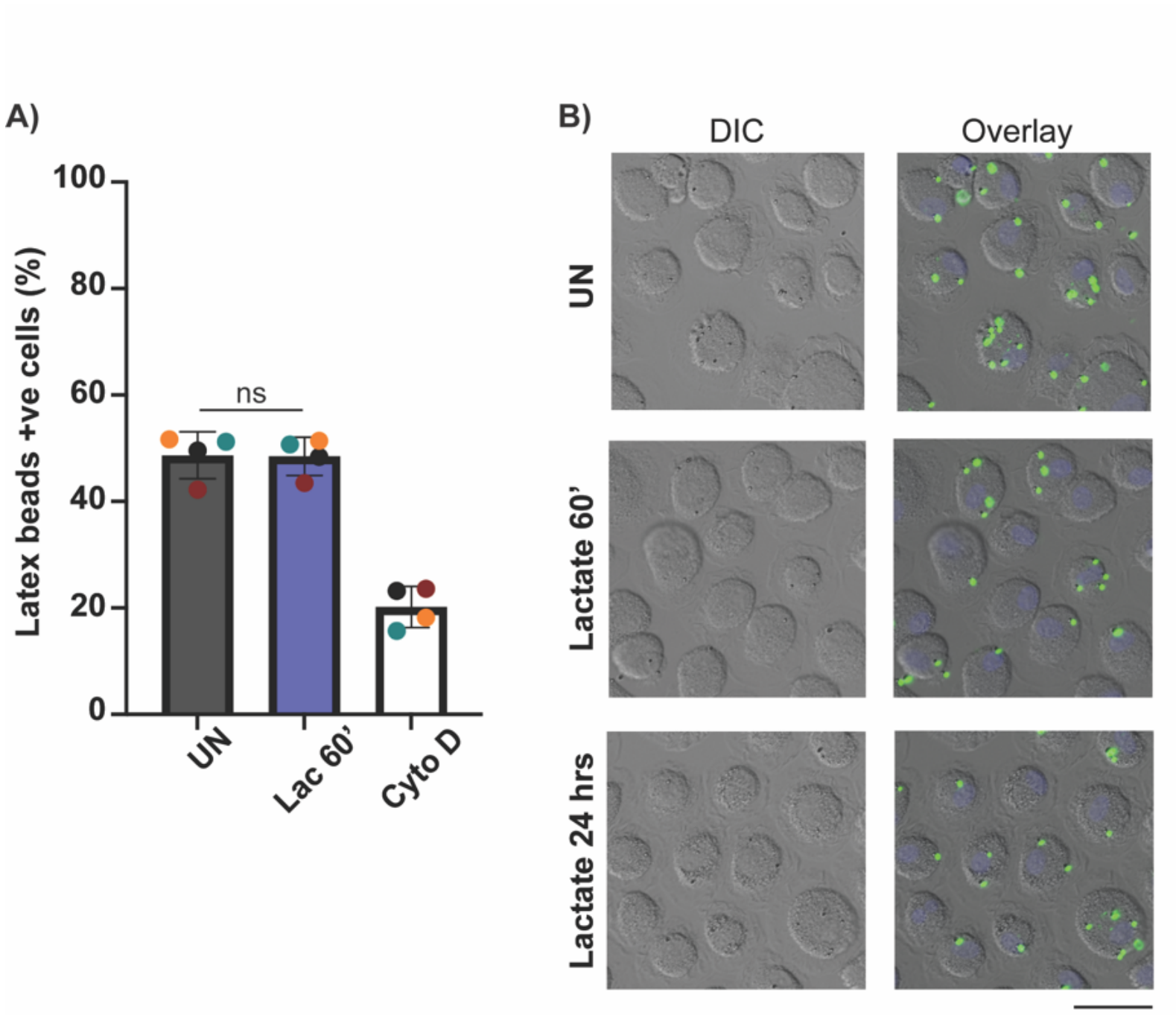

Figure S2: (A) Mφ with or without lactate treatment were incubated with fluorescent 1 μm latex beads at an MOI of 5 for 30 minutes, and bead uptake was analyzed using a Cytoflex flow cytometer. Mφ treated with 10 μM cytochalasin D was used as a negative uptake control. Each color denotes a donor. Statistics: linear mixed effects model, donor as a random effect, and treatment as a fixed effect. (B) Mφ were treated with lactate, exposed to latex beads, fixed using 4% paraformaldehyde, and microscopically visualized. Representative data is shown. Scale bar 100 μm.

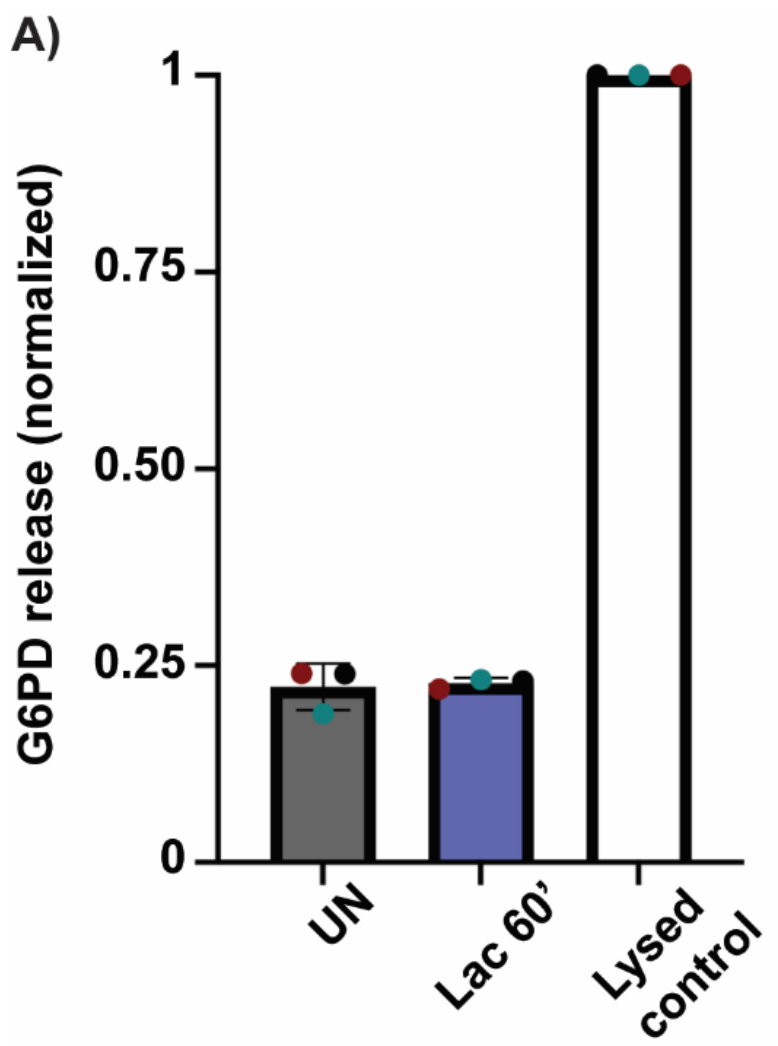

Figure S3: (A) Mφ supernatant was collected following lactate treatment, and glucose-6-phosphate dehydrogenase (G6PD) release was quantified as a measure of cellular cytotoxicity. Each color denotes a donor. Statistics: linear mixed effect model, donor as a random effect, and treatment as a fixed effect.

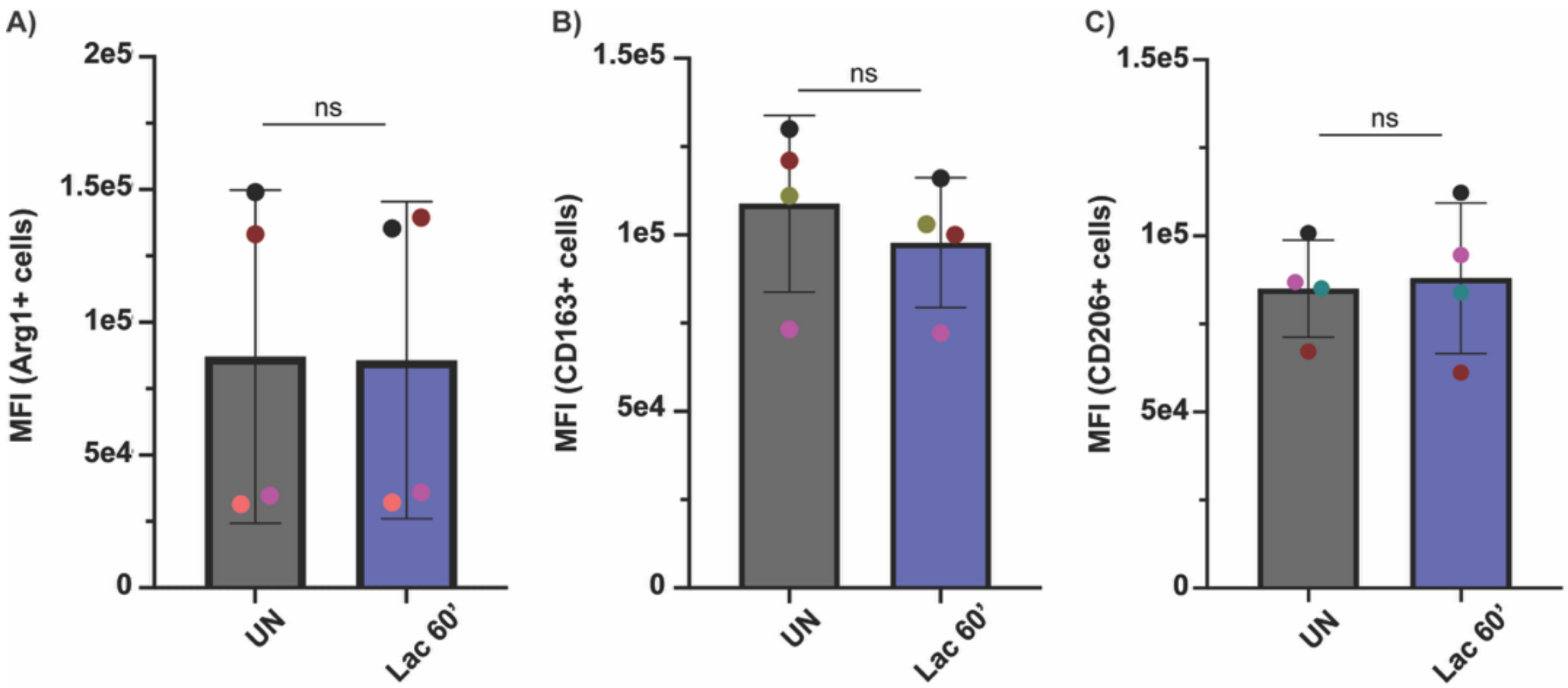

Figure S4: Mφ intracellular (Arg1, A) and cell surface (CD163 and CD206, B & C, respectively) expression in mean fluorescent intensity (MFI) for the control or 10 mM lactate treatment (*MFI data from Fig 4B*). At least 10000 events were analyzed per condition using the CytoFlex flow cytometer, and Mφ positive for CD14 and respective markers were analyzed using FlowJo 10.10.0 software. Each color denotes a donor.

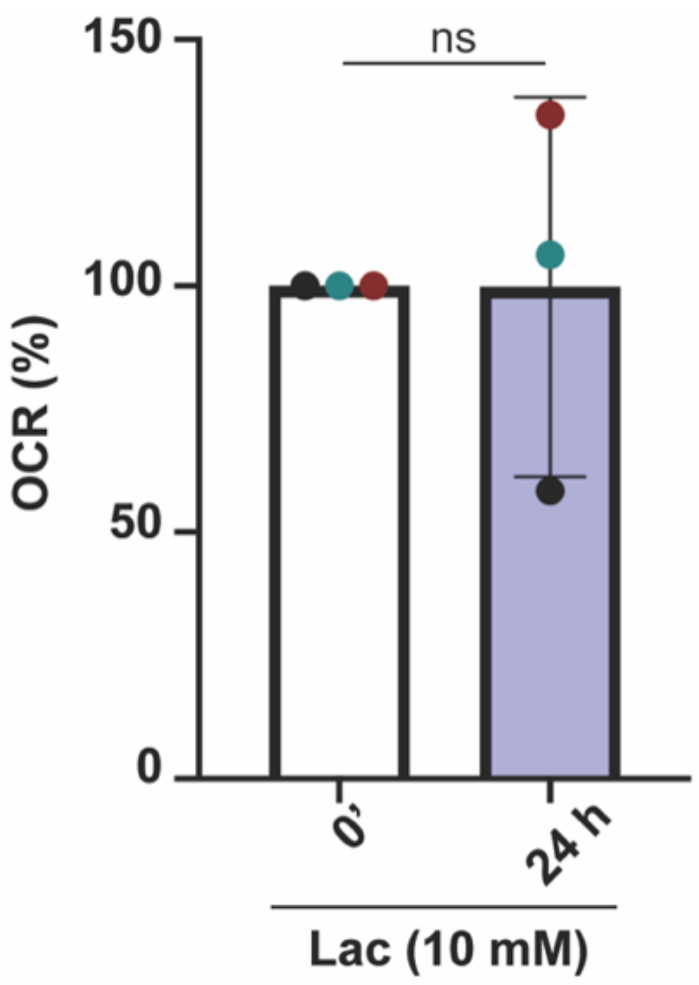

Figure S5: Mφ was exposed to 10 mM of lactate for 24 hrs and oxygen consumption rate (OCR) was spectrophotometrically quantified using MitoXpress Xtra probe. Each color denotes a donor. Statistics: linear mixed effect model, donor as a random effect, and treatment as a fixed effect.
